# SPI1-mediated MIR222HG transcription promotes proneural-to-mesenchymal transition of glioma stem cells and immunosuppressive polarization of macrophages

**DOI:** 10.7150/thno.82590

**Published:** 2023-05-27

**Authors:** Yang Fan, Zijie Gao, Jianye Xu, Huizhi Wang, Qindong Guo, Boyan Li, Ming Li, Hao Xu, Yanhua Qi, Shulin Zhao, Wei Qiu, Ziwen Pan, Qingtong Wang, Hao Xue, Rongrong Zhao, Xing Guo, Gang Li

**Affiliations:** 1 Department of Neurosurgery, Qilu Hospital, Cheeloo College of Medicine and Institute of Brain and Brain-Inspired Science, Shandong University, Jinan 250012, Shandong, China.; 2 Shandong Key Laboratory of Brain Function Remodeling, Jinan 250012, Shandong, China.; 3 Tianjin Neurological Institute, Key Laboratory of Post-Neuroinjury Neuro-repair and Regeneration in Central Nervous System, Ministry of Education and Tianjin City, Tianjin Medical University General Hospital, Tianjin 300052, China.; 4 Department of Neurosurgery, The Affiliated Taian City Central Hospital of Qingdao University, Taian 271000, Shandong, China.; 5 Department of Neurosurgery, The Affiliated Yantai Yuhuangding Hospital of Qingdao University, Yantai 264000, Shandong, China.

**Keywords:** Glioma stem cell, HDAC, Mesenchymal transition, Radiation, Immunosuppressive

## Abstract

**Background:** Glioma stem cells (GSCs) are a key factor in glioblastoma (GBM) development and treatment resistance. GSCs can be divided into the mesenchymal (MES) and proneural (PN) subtypes, and these two subtypes of GSCs can undergo interconversion under certain conditions. MES GSCs have higher malignancy and radioresistance and are closely associated with an immunosuppressive microenvironment. Long noncoding RNAs (lncRNAs) play a broad role in GBM, while the role of GSCs subtype remains unknown.

**Methods:** We performed RNA sequencing to explore the lncRNA expression profile in MES- and PN-subtype GBM tissues. The biological function of a host gene—MIR222HG—in GBM development was confirmed *in vitro* and *in vivo*. Specifically, RNA sequencing, RNA pulldown, mass spectrometry, RIP, ChIP, luciferase reporter assays and Co-IP were performed.

**Results:** MIR222HG, the expression of which can be induced by SPI1, has high levels in MES GBM tissues. Functionally, we demonstrated that MIR222HG promotes the MES transition and radioresistance in GSCs *in vivo* and *in vitro*. Mechanistically, MIR222HG can bind to the YWHAE/HDAC5 complex to promote the MES transition of GSCs through H4 deacetylation. Moreover, cotranscribed miR221 and miR222 can be delivered to macrophages via exosomes to target SOCS3, causing immunosuppressive polarization. Finally, PLX-4720 sensitivity is associated with SPI1 expression and acts on MES GSCs to enhance radiosensitivity.

**Conclusions:** This study demonstrates that targeting SPI1 to block transcription of the MIR222HG cluster helps to reduce radioresistance and combat the immunosuppressive microenvironment in GBM. PLX-4720 is a potential GBM drug and radiosensitizer.

## Introduction

Glioblastoma (GBM) is the most common intracranial primary malignant tumor, with high therapeutic resistance and a poor prognosis 1-3. The high degrees of intratumoral cellular heterogeneity and plasticity are the main reasons for the poor prognosis of GBM patients 4. Currently, according to the characteristics of GBM gene expression profiles in The Cancer Genome Atlas (TCGA), GBM is divided into three tumor-intrinsic transcriptional subtypes: classical (CL), proneural (PN) and mesenchymal (MES) 5. Among them, MES GBM has higher radioresistance and poorer prognosis and is associated with recurrence after treatment 6, 7. GSCs are stem cell-like tumor-initiating cells with self-renewal ability and multilineage differentiation potential 8, 9. At the extremes of the axis of genetic variation in GBM are stem-like cells that express canonical markers of MES GSCs (mGSCs) and those that express canonical markers of PN GSCs (pGSCs) 10. mGSCs and pGSCs are stable *in vitro*, representing the heterogeneous and plastic characteristics of GBM. mGSCs have a higher proliferative capacity, greater tumorigenicity, and higher resistance to radiotherapy and harsh microenvironments than pGSCs 7, 11, 12. Proneural-to-mesenchymal transition (PMT) in GSCs is a main cause of radioresistance and tumor recurrence during the malignant progression of GBM 13. Our previous study indicated that macrophage-derived small extracellular vesicles (sEVs) are key regulators of PMT in GSCs and that upregulation of cell surface GRP78 in mGSCs plays a critical role in maintaining the MES subtype 14, 15. Although numerous studies have explored the evolution of PMT, the molecular mechanisms underlying PMT in GSCs remain unknown.

The tumor microenvironment (TME) is the foundation for tumor growth and is composed of immune cells, stromal cells, the extracellular matrix (ECM), secreted molecules and other components 16. The TME is closely related to the molecular phenotypic heterogeneity of GBM and affects the regulation of the mechanism underlying the molecular phenotypic plasticity of GBM 17, 18. In the TME of GBM, tumor-associated macrophages (TAMs) are the most abundant cell population, accounting for 50% of the total cells in the GBM TME, and play an important role in the interactive ecology of the immunosuppressive TME and malignant progression of GBM 19. Exosomes are extracellular secretory vesicles with a diameter of 50-100 nm that can encapsulate proteins, RNAs and other substances for transfer between cells 20. Tumor cells and macrophages can interact with each other via exosome delivery 21, 22. Our previous study demonstrated that sEVs, including exosomes, can be transferred from immunosuppressive macrophages into GSCs to induce PMT; however, whether mGSCs can also act in reverse to induce macrophage immunosuppression remains to be explored 14.

Noncoding RNAs, including microRNAs (miRNAs) and long noncoding RNAs (lncRNAs), are a class of RNAs that cannot encode proteins and play important roles in tumors 23. An increasing number of studies have shown that lncRNA expression profiles can reflect the intrinsic PN and MES subtype characteristics of GSCs, but whether lncRNAs are involved in PMT in GSCs has not been explored 24, 25.

In this study, we identified the highly expression of MIR222HG in the MES subtype by comparing the differentially expressed lncRNAs between the PN and MES subtypes of GBMs and GSCs. MIR222HG, a lncRNA produced from a miRNA host gene (Lnc-MIRHG), can generate multiple different lncRNA transcripts as well as miRNA-221/222 (miR221/222) through cotranscriptional splicing. Among these products, the most abundant lncRNA transcript, MIR222HG-201, recruits the histone deacetylase 5 (HDAC5)/YWHAE complex by anchoring histone H4 in the nucleus of GSCs, mediates the deacetylation of H4K16, leads to PMT in GSCs, and then enhances radioresistance. The cotranscribed miR221 and miR222 can be transferred from GSCs to macrophages via exosomes, mediating a decrease in SOCS3 expression and leading to immunosuppressive polarization of macrophages. As a transcription factor for the MIR222HG gene, SPI1 mediates the cotranscription of lncRNAs and miRNAs, resulting in PMT in GSCs and immunosuppressive polarization of macrophages. Finally, we used PLX-4720 to screen for GBM cell sensitivity associated with SPI1 expression. These experiments confirmed that mGSCs are sensitive to PLX-4720 and that its combined use with radiotherapy can significantly enhance the therapeutic efficacy.

## Materials and methods

### Patient specimens and data acquisition

Twelve human GBM tissues were obtained for RNA sequencing (RNA-seq) from patients admitted to Qilu Hospital between November 2017 and October 2019. All participants provided written informed consent, and the study was approved by the Ethics Committee on Scientific Research of Shandong University Qilu Hospital (approval number: KYLL-2018-324).

The RNA-seq transcriptome data for the GBM dataset in The Cancer Genome Atlas (TCGA) were obtained from the TCGA database (http://cancergenome.nih.gov/). The Chinese Glioma Genome Atlas (CGGA) RNA-seq dataset was obtained from the CGGA database (http://www.cgga.org.cn/). The European Nucleotide Archive (ENA) RNA-seq dataset was obtained from the ENA database (PRJEB27943; http://www.ebi.ac.uk/ena). The RNA-seq data of our local samples have been deposited in the Gene Expression Omnibus (GEO) under accession number GSE211554. The RNA-seq data of GSC267 have been deposited in GEO under accession number GSE213310.

### Cell lines and culture

The patient-derived GSC cell lines (GSC11, GSC8-11, GSC20, GSC28, and GSC267) and neural progenitor cells (NPCs) were a kind gift from Dr. Frederick F. Lang and Dr. Krishna P.L. Bhat (M.D. Anderson Cancer Center, University of Texas, Houston, TX). The subtypes of GSCs in this study were identified according to the genetic signatures of Philips and Verhaak, which are widely accepted and applied 7, 11. GSCs and NPCs were digested into single cells with Accutase solution (Sigma‒Aldrich, USA) and were then cultured in DMEM/F12 (Gibco, USA) supplemented with B-27 (Gibco, USA), 20 ng/mL recombinant human (rh) epidermal growth factor (R&D Systems, USA) and 20 ng/mL rh basic fibroblast growth factor (R&D Systems, USA). Cells were cultured at 37 °C in a humidified chamber with 5% carbon dioxide and 5% oxygen.

### Antisense oligonucleotide (ASO), small interfering RNA (siRNA), miRNA mimic (miRNAm) and miRNA inhibitor (miRNAi) transfection and lentiviral transduction

The ASO, siRNA, miRNAm, miRNAi and corresponding negative controls were purchased from GenePharma (China). The knockdown and overexpression lentiviruses and corresponding negative control lentiviruses were synthesized by GeneChem (Shanghai, China). All sequence information is provided in Table S8. Cells were transfected using a Lipofectamine 3000 kit (Invitrogen, Carlsbad, CA, USA) according to the manufacturer's instructions.

### Ionizing radiation (IR) treatment

*In vitro*, GSCs were treated with IR (6 Gy) for subsequent experiments. *In vivo*, tumor-bearing mice were administered 4 doses of IR (2.5 Gy each) within 8 to 12 days after GSC implantation.

### Real-time quantitative PCR (qRT‒PCR)

Total RNA was extracted with TRIzol (Invitrogen, USA) following the manufacturer's protocol. We performed reverse transcription using a High-Capacity-cDNA Reverse Transcription Kit (Toyobo, China) according to the manufacturer's protocol. The PCR primer pair sequences are listed in the supplementary materials and methods, and an Mx-3000P Quantitative PCR System (Applied Biosystems, USA) was used for qRT‒PCR. The detailed sequences of the primers are listed in Table S7.

### Fluorescence *in situ* hybridization (FISH)

The FISH assay was performed as previously described 26. The RNA FISH probe was designed and synthesized by GenePharma (Shanghai, China). The probe sequences are listed in Table S7.

### Nuclear-cytoplasmic fractionation

A PARIS™ kit (Thermo Fisher Scientific, USA) was used according to the manufacturer's instructions to perform nuclear-cytoplasmic fractionation. RNA was extracted from the nuclear and cytoplasmic fractions of cells (1 × 10^7^) and was then analyzed by qRT‒PCR.

### Neurosphere formation assay

GSCs were dispersed with Accutase into single cells and seeded into 6-well plates at a density of 1000 cells per well. After 2 weeks of culture in GSC culture medium, cells were observed using a Leica microscope, and the relative diameters of spheres were recorded for analysis.

### Extreme limiting dilution assay (ELDA)

GSCs were seeded into 96-well plates in a concentration gradient of 0, 2, 4, 8, 16, 32 and 64 cells. Ten replicates per well were established. The numbers of wells with tumor sphere formation were recorded after one week, and the data were analyzed with ELDA software (http://bioinf.wehi.edu.au/software/elda/).

### Western blot analysis

Precipitated cells were washed with cold PBS and lysed with RIPA buffer containing 1% protease and phosphate inhibitor cocktail (P8340; Sigma‒Aldrich, USA). After sodium dodecyl sulfate-polyacrylamide gel electrophoresis (SDS‒PAGE), proteins were transferred to polyvinylidene fluoride (PVDF) membranes. Then, the membranes were cut into strips and incubated with primary antibodies at 4 °C overnight and were then incubated with secondary antibodies. We examined protein expression using an Odyssey fluorescence scanner (ChemiDoc XRSþ, Bio-Rad). All antibody information is provided in Table S9.

### Apoptosis assay

We used a FITC-Annexin V apoptosis detection kit (556, 547; BD Biosciences, USA) according to the manufacturer's protocol to determine the proportion of apoptotic cells. The data were generated with a BD Accuri C6 flow cytometer and further processed with FlowJo V10.

### Cell cycle analysis

After treatment, GSC neurospheres were collected by centrifugation and dissociated with Accutase solution. Then, we resuspended and stained the GSCs with propidium iodide (PI) staining solution (BD Pharmingen). Finally, we conducted cell cycle analysis using a BD Accuri C6 flow cytometer (BD Biosciences).

### Alkaline comet assay

We performed an alkaline comet assay (4250-050-K; R&D Systems, USA) following the manufacturer's instructions to evaluate DNA damage levels. In brief, GSCs subjected to different treatments were collected and harvested in PBS at a density of 1 × 10^5^ cells/mL. We then mixed the GSCs with low-melting-point (LM) agarose at a ratio of 1:10 (V/V) and immediately added 50 µL of the mixture onto a comet slide. The cells were further lysed with alkaline lysis buffer for 12 h at 4 °C, and the slides were soaked in alkaline electrophoresis solution for 20 min before electrophoresis at 25 V for 30 min. Finally, we stained the slides with SYBR Green DNA Dye and acquired images with a fluorescence microscope.

### Terminal deoxynucleotidyl transferase dUTP nick end labeling (TUNEL) assay

The TUNEL assay was conducted using a TUNEL apoptosis assay kit (C1090; Beyotime, China) according to the manufacturer's instructions. DAPI solution was required for nuclear staining (C1006; Beyotime, China). The proportion of apoptotic cells (red fluorescence) was determined and images were acquired using a Lecia microscope.

### Biotin-labeled RNA pulldown assay and mass spectrometry

Lnc-MIR222HG-201 and its antisense sequence were synthesized by RiboBio (GenePharma, Shanghai, China). The assays were performed as previously described 26. Finally, the interacting proteins extracted from GSC267 cells were identified by western blotting and mass spectrometry.

### Coimmunoprecipitation (Co-IP)

We conducted a Co-IP assay using a Pierce Classic Magnetic IP/Co-IP Kit (Thermo Fisher, USA) according to the manufacturer's protocol. In brief, we incubated antibodies with protein A/G magnetic beads. Then, we obtained GSC267 lysates and mixed them with antibody-coupled beads overnight at 4 °C. After washing and denaturation, proteins interacting with the beads were collected for western blotting. All antibody information is provided in Table S9.

### RNA immunoprecipitation (RIP)

RIP assays were performed according to the instructions of an EZ-Magna RIP RNA-binding Protein Immunoprecipitation Kit (Merck Millipore). The extracted RNA was analyzed by qRT-PCR. For the YWHAE RIP assay, wild-type (WT) cDNA and mutant (mut) cDNA were subcloned into the pcDNA3.1 vector (BioSune, China). All antibody information is provided in Table S9.

### Exosome isolation and identification

Exosomes were isolated from GSC culture supernatant using several centrifugation and ultracentrifugation steps as previously described and were stored at -80 °C 27, 28. Transmission electron microscopy (TEM) was used to photograph exosome morphology, and a ZetaView system (Particle Metrix, Germany) was used to measure the exosome particle size and concentration as previously described 28, 29. Western blot analysis was used to detect markers to distinguish between exosomes and cells. Purified exosomes were labeled with the green fluorescent linker PKH67 (Sigma‒Aldrich, USA) and used for uptake experiments as previously described 28, 29.

### Flow cytometry

Anti-CD163-PE and anti-CD11b-APC antibodies were used for staining to detect CD11b^+^CD163^+^ macrophages. Isotype controls were run in parallel. Flow cytometry was performed by using a BD Accuri C6 flow cytometer (BD Biosciences). Antibody information is provided in Table S9.

### Luciferase reporter assay

The reporter plasmids PmirGLO-SOCS3-3'UTR-WT and PmirGLO-SOCS3-3'UTR-mut were cotransfected with the miR221 mimic or miR222 mimic into THP1 differentiated macrophages. The reporter plasmids pGL3-MIR222HG-WT and pGL3-MIR222HG-mut were cotransfected with siSPI1#1 or siSPI1#2 into GSC267 cells. Two days later, a dual luciferase reporter assay kit (Promega, USA) was used to measure reporter activity according to the manufacturer's instructions. All reporter plasmids were synthesized by BioSune (Shanghai, China).

### Chromatin immunoprecipitation (ChIP)

ChIP was performed with an EZ-Magna ChIP A/G Chromatin Immunoprecipitation Kit (17-10086, Millipore, USA). Immunoprecipitated DNA fragments were quantified with qRT‒PCR. The primers and antibodies used are listed in Tables S7 and S9.

### Cell counting kit-8 (CCK-8) assay

We purchased PLX-4720 from MedChemExpress (MCE; https://www.medchemexpress.cn/). PLX-4720 dissolved in dimethyl sulfoxide (DMSO) was stored at -20 °C and used within one month. GSC20 and GSC267 cells were seeded into 96-well plates at a density of 5000 cells per well, treated with different concentrations of PLX-4720, and cultured at 37 °C for 48 h. Then, we added 10 μL of CCK-8 solution (Beyotime, China) to each well, and the absorbance, reflecting cell proliferation, was measured 1 h later.

### *In vivo* experiments

We generated GSC267 and GSC8-11 cells labeled with luciferase (GSC267-luciferase, GSC8-11-luciferase) via lentiviral transduction. All animal experiments were performed with approval according to the guidelines of the Institutional Animal Care and Use Committee of Qilu Hospital of Shandong University. Four-week-old male BALB/c nude mice (SLAC Laboratory Animal Center; Shanghai, China) were bred under specific pathogen-free conditions at 24 °C on a 12-h day-night cycle in preparation for the establishment of an intracranial GSC *in situ* growth model. We randomly divided animals housed under similar conditions into the control and experimental groups. A total of 1 × 10^6^ GSC267-luciferase or GSC8-11-luciferase cells were injected intracranially into each mouse. When irradiation was necessary in animal studies, tumor-bearing mice were administered 4 doses of IR (2.5 Gy each) within 8 to 12 days after GSC implantation. Mice in the dosing group were injected daily with PBS or an equal volume of PLX-4720 (15 mg/kg) via the tail vein beginning 7 days after GSC implantation. Tumor progression *in vivo* was measured by bioluminescence imaging after intraperitoneal injection of 150 mg/kg luciferin; signals were detected and images were acquired with an IVIS Lumina series III *ex vivo* imaging system (PerkinElmer, USA).

### Hematoxylin and eosin (HE) staining and immunohistochemical (IHC) staining

HE and IHC staining were performed as previously described 29. All information about the primary antibodies used for IHC staining is provided in Table S9.

### Gene set enrichment analysis (GSEA)

The Phillips/Verhaak glioblastoma mesenchymal and Phillips/Verhaak glioblastoma proneural gene signatures were obtained from the Molecular Signatures Database (MSigDB; http://www.gsea-msigdb.org/gsea/login.jsp). GSEA_4.1.0 software was used for GSEA.

### Single-cell RNA-seq analysis

We downloaded single-cell RNA-seq data from the GEO (https://www.ncbi.nlm.nih.gov/geo/, GSE138794). We further performed t-distributed stochastic neighbor embedding (tSNE) to identify different cell clusters using the R package “Seurat 4.1.0” and evaluated the enrichment scores of the Verhaak_GBM_MES signature based on the R package “irGSEA”.

### Association of SPI1 expression with drug sensitivity

The drug sensitivity data and RNA transcriptome data for GBM cell lines were downloaded from the Genomics of Drug Sensitivity in Cancer (GDSC) database (www.cancerRxgene.org) and the Cancer Cell Line Encyclopedia (CCLE; https://portals.broadinstitute.org/ccle/), respectively. Then, we performed Spearman correlation analysis to identify the drugs that were significantly associated with SPI1 expression.

### Statistical analysis

We used GraphPad Prism 8.0 software and R 4.0.1 to perform all statistical analyses. Student's t test and one-way ANOVA were performed to compare differences between two groups and among more than two groups, respectively. We performed Pearson correlation analysis to evaluate correlations between different groups. For survival analysis, Kaplan‒Meier (KM) curves were generated and the log-rank test was performed to visualize and compare survival between different groups, respectively. P values of < 0.05 were accepted as statistically significant (*P value < 0.05; **P value < 0.01; ***P value < 0.001).

## Results

### MIR222HG enhances GSC self-renewal and mediates the PMT process

**Figure 1:**
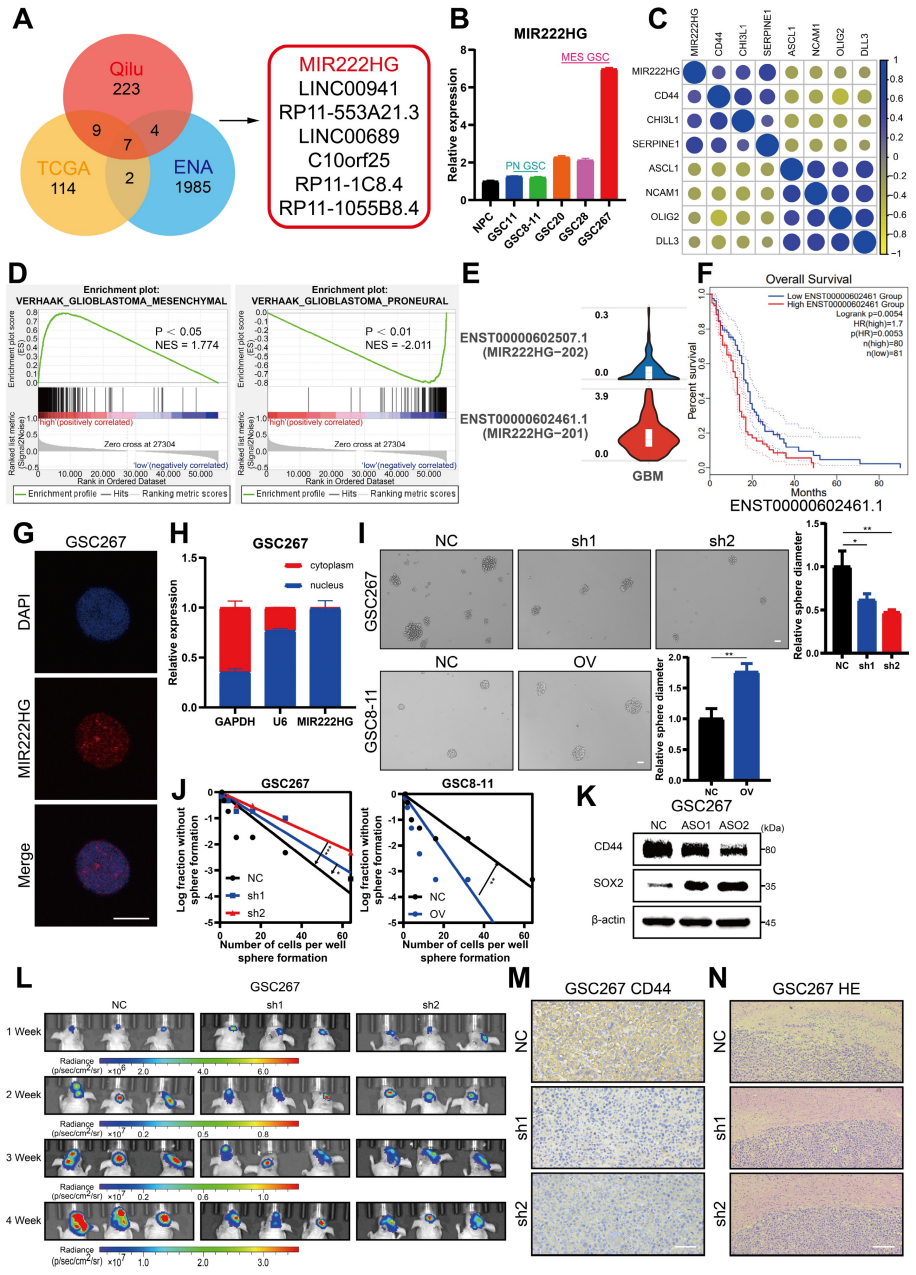
** MIR222HG enhances GSC self-renewal and mediates the PMT process. A** Venn diagram showing the overlap of lncRNAs upregulated in the MES subtype identified in the TCGA dataset, ENA dataset and Qilu dataset. **B** qRT‒PCR showing the relative expression of MIR222HG in NPCs, pGSCs (GSC11 and GSC8-11) and mGSCs (GSC20, GSC28, and GSC267). **C** Correlations of MIR222HG expression with the expression of PN subtype marker genes (DLL3, OLIG2, ASCL1, NCAM1) and MES subtype marker genes (CD44, CHI3L1 and SERPINE1). **D** GSEA based on the Verhaak gene set showed that MIR222HG expression was positively correlated with the MES subtype and negatively correlated with the PN subtype. **E** Relative expression of the two transcripts of MIR222HG in GBM. **F** Kaplan-Meier curves revealing the overall survival of GBM patients stratified according to MIR222HG expression.** G** RNA FISH assays showing the subcellular localization of MIR222HG (Cy3) in GSC267 cells. Nuclei were stained with DAPI (blue). Scale bar, 15 μm. **H** Nuclear-cytoplasmic fractionation assays showing the relative expression of MIR222HG in the cytoplasmic (GAPDH) and nuclear (U6) fractions. **I** Representative images and quantification of tumor sphere formation in GSC8-11 cells with MIR222HG overexpression and in GSC267 cells with MIR222HG knockdown. Scale bar, 50 μm.** J** ELDA of GSC267 cells expressing lentiviral shNC or shMIR222HG and GSC8-11 cells expressing lentiviral vector or MIR222HG. **K** western blot analysis of CD44 and SOX2 protein expression after knockdown of MIR222HG in GSC267 cells. β-Actin served as the negative control.** L** Bioluminescence imaging to measure tumor sizes in shNC, sh1 and sh2 GSC267 xenograft-bearing mice.** M** Representative images of IHC staining for CD44 in sections of GSC267 xenografts from each group. Scale bar, 25 μm. **N** Representative images of HE staining of tissue from a subgroup of animals in each group sacrificed simultaneously. Scale bar, 200 μm. All data are presented as the means ± SDs. ns, P > 0.05; *P < 0.05; **P < 0.01; ***P < 0.001.

To explore the lncRNAs potentially regulating PMT, we used ssGSEA to calculate MES and PN scores using the Phillips gene set in sequencing data of 12 GBM samples from the neurosurgery department of Qilu Hospital (GEO: GSE211554) and divided the samples into two groups—namely, High-Score and Low-Score—for difference analysis. The differentially expressed genes were then intersected with the differentially expressed genes in MES GBM and PN GBM in TCGA and the differentially expressed lncRNAs in MES GSCs and PN GSCs in the ENA (PRJEB27943). Seven lncRNAs with high expression in the MES subtype were identified (Figure 1A and Figure S1A). We applied qRT‒PCR to measure the expression of these 7 lncRNAs in NPCs and GSC cell lines (pGSC: GSC11, GSC8-11; mGSC: GSC20, GSC28, GSC267) from the MD Anderson Cancer Center (Figure 1B and Figure S1C). MIR222HG was the most consistent with the expectations, with higher expression in MES GSCs than in PN GSCs and higher expression in GSCs than in NPCs. Subsequently, we performed a preliminary bioinformatic analysis of MIR222HG. Correlation analysis based on TCGA data revealed that the expression of MIR222HG was positively correlated with that of MES subtype marker genes (CD44, CHI3L1 and SERPINE1) and negatively correlated with that of PN subtype marker genes (DLL3, OLIG2, ASCL1, NCAM1) (Figure 1C and Table S1). Single-cell RNA-seq analysis showed that the clusters of cells with high MIR222HG expression had higher MES scores (Figure S1B). GSEA based on the Verhaak and Phillips gene sets showed that high expression of MIR222HG was more prevalent in the MES subtype, and low expression of MIR222HG was more prevalent in the PN subtype (Figure 1D and Figure S1D). In further studies, we found that MIR222HG was expressed in GBM mainly as two transcripts, MIR222HG-201 and MIR222HG-202; the MIR222HG-201 transcript was the most highly expressed, and we thus concluded that it was the MIR222HG-201 transcript representing the role of MIR222HG (Figure 1E). Survival analysis indicated poor prognosis for patients with high expression of MIR222HG-201 and good prognosis for those with low expression (Figure 1F). We confirmed by FISH and nuclear-cytoplasmic fractionation that MIR222HG is a lncRNA localized in the nucleus (Figure 1G, H). We first used an ASO to knock down MIR222HG in mGSCs and found a significant reduction in the expression of the MES marker CD44 and a significant increase in that of the PN marker SOX2 by western blot analysis. pGSCs with overexpression of MIR222HG in exhibited a significant reduction in SOX2 expression. We did not detect CD44 in GSC8-11 cells, which is consistent with the lack of CD44 expression in GSC8-11 cells (Figure 1K and Figure S1I). We subsequently generated lentivirally transduced cell lines. The neurosphere formation assay and ELDA showed that knockdown of MIR222HG in mGSCs significantly reduced their self-renewal capacity, whereas overexpression of MIR222HG in pGSCs enhanced their self-renewal capacity (Figure 1I, J and Figure S1E, F). This pattern provides evidence that MIR222HG promotes the malignant progression of GBM by enhancing the self-renewal capacity of GSCs. *In vivo*, through a xenograft model, knockdown of MIR222HG was demonstrated to significantly reduce the tumorigenesis of GSC267 cells and prolong the survival of mice (Figure 1L and Figure S1G, H). IHC staining of tumor sections showed a significant decrease in CD44 expression, while HE staining showed a significant decrease in tumor aggressiveness (Figure 1M, N). In contrast, overexpression of MIR222HG enhanced the tumorigenesis and aggressiveness of GSC8-11 cells and reduced survival and SOX2 expression in mice (Figure S1J, K, L, M, N). In summary, we found that the nuclear transcript MIR222HG-201 is highly expressed in the MES subtype, mediates the PMT process, and enhances the self-renewal capacity and tumorigenicity of GSCs.

### MIR222HG expression correlates with radioresistance in mGSCs

**Figure 2:**
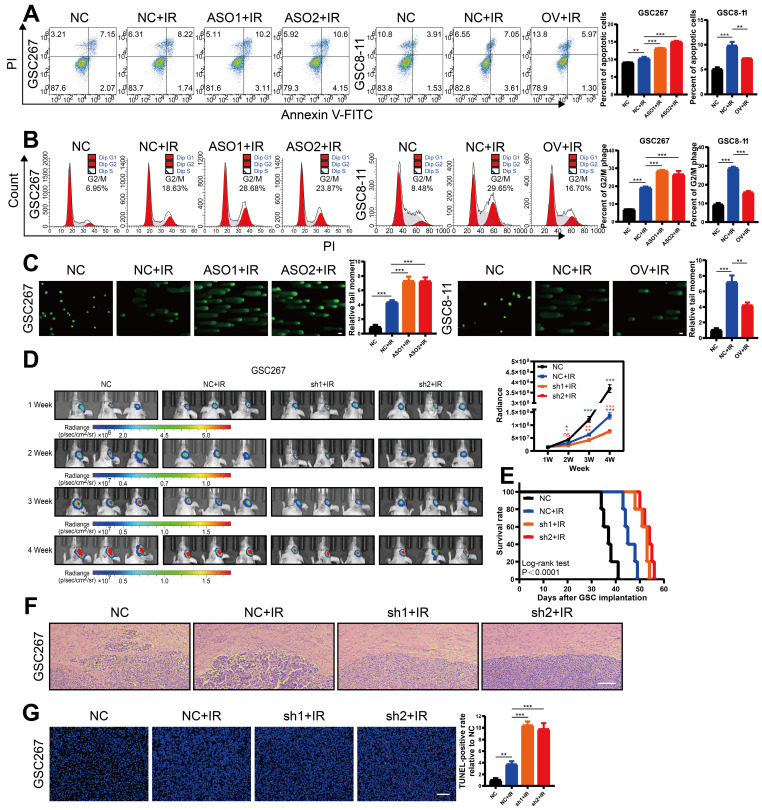
** MIR222HG expression correlates with radioresistance in mGSCs. A, B, C** The effects of knocking down MIR222HG in GSC267 cells and overexpressing MIR222HG in GSC8-11 cells on the outcomes of IR (6 Gy) treatment were evaluated by apoptosis **(A)**, cell cycle **(B)** and alkaline comet **(C)** assays. The corresponding quantifications are shown on the right. Scale bar, 25 μm. **D** Bioluminescence images (left) and quantification (right) of tumor size in shNC, sh1 and sh2 GSC267 xenograft-bearing nude mice receiving IR treatment. **E** Kaplan-Meier curves showing the survival of GSC267 xenograft-bearing mice in the different groups. **F** Representative images of HE staining of tissues from a subgroup of animals in each group sacrificed simultaneously. Scale bar, 200 μm. **G** Representative images and quantification of TUNEL staining in sections of IR-treated GSC267 xenografts. Scale bar, 200 μm. All data are presented as the means ± SDs. ns, P > 0.05; *P < 0.05; **P < 0.01; ***P < 0.001.

GBM patients derive little benefit from radiotherapy, mainly owing to the radioresistance of mGSCs 7. Here, we further investigated whether the MIR222HG-mediated MES subtype orchestrates the acquisition of radioresistance in GSCs. GSCs exhibit DNA damage, G2/M arrest and apoptosis after radiation treatment. Here, mGSCs exhibited less apoptosis, G2/M arrest and DNA damage after radiation treatment than pGSCs. Furthermore, knockdown of MIR222HG in mGSCs enhanced the response to radiotherapy. In contrast, overexpression of MIR222HG in pGSCs attenuated the response to radiotherapy (Figure 2A, B, C and Figure S2A, B, C). *In vivo*, knockdown of MIR222HG in mGSCs enhanced the radiotherapy response, attenuated tumorigenesis, and prolonged survival in mGSC-bearing mice (Figure 2D, E). HE staining of mouse brain sections revealed that knockdown of MIR222HG combined with radiotherapy significantly reduced tumor aggressiveness (Figure 2F). TUNEL assays on tissue sections demonstrated that knockdown of MIR222HG enhanced apoptosis in mGSCs *in vivo* (Figure 2G). In contrast, mice implanted with pGSCs overexpressing MIR222HG showed reduced responsiveness to radiotherapy, faster tumor growth and shorter survival after radiotherapy (Figure S2D, E). HE staining revealed a significant increase in tumor aggressiveness in the group implanted with pGSCs overexpressing MIR222HG and treated with radiotherapy (Figure S2F). The TUNEL assay demonstrated that overexpression of MIR222HG reduced the apoptosis level in tumors *in vivo* (Figure S2G). In summary, MIR222HG enhanced the radioresistance of mGSCs both *in vivo* and *in vitro*.

### MIR222HG induces PMT by mediating H4K16 deacetylation

**Figure 3:**
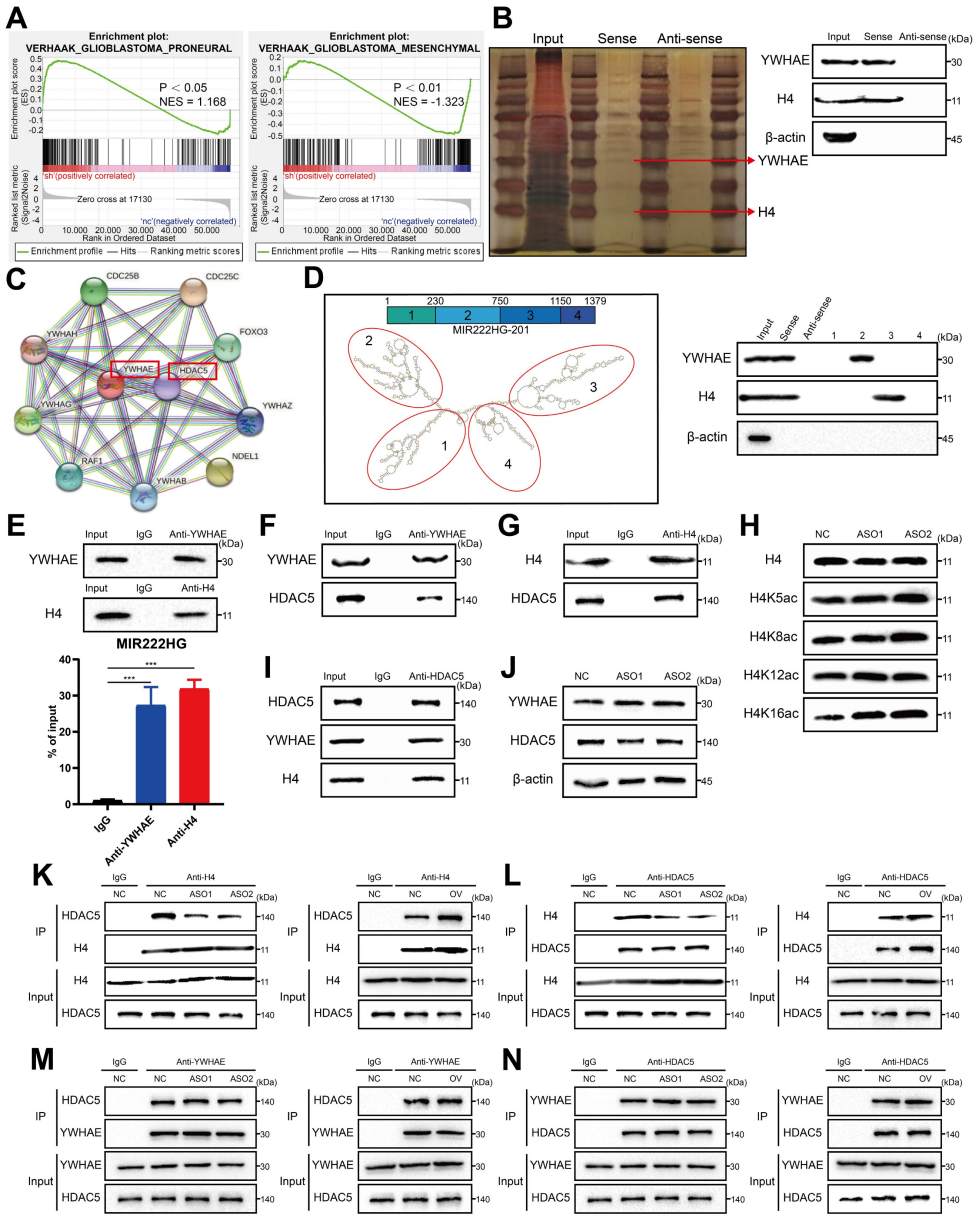
** MIR222HG induces PMT by mediating H4K16 deacetylation. A** GSEA based on the Verhaak gene set showed that knockdown of MIR222HG expression in GSC267 cells was negatively correlated with the MES subtype and positively correlated with the PN subtype. **B** Silver staining assay showing the proteins that interacted with MIR222HG, which were identified by RNA pulldown/mass spectrometry (left). Western blot analysis showing the interaction between MIR222HG and YWHAE or H4 in GSC267 cells (right). **C** The binding of YWHAE to HDAC5 was predicted by STRING. **D** The secondary structure of MIR222HG was predicted with the RNAfold WebServer. An RNA pulldown assay was performed with *in vitro* transcribed biotinylated RNAs corresponding to different fragments of MIR222HG in GSC267 cells. **E** RIP-qPCR assay showing the relative enrichment of MIR222HG in GSC267 cells as detected by anti-YWHAE and anti-H4 antibodies (bottom). Specific immunoprecipitation of YWHAE and H4 was confirmed by western blot analysis (top). **F, G** The interactions of YWHAE with HDAC5 and H4 with HDAC5 in GSC267 cells were detected by Co-IP with anti-YWHAE **(F)** and anti-H4 **(G)** antibodies, respectively. **H** western blot showing the extent of acetylation of each H4 site in GSC267 cells after knockdown of MIR222HG with an ASO. **I** The interactions of HDAC5 with YWHAE and H4 in GSC267 cells were detected by Co-IP with an anti-HDAC5 antibody. **J** western blot was performed to detect changes in the expression of YWHAE and HDAC5 in GSC267 cells after knockdown of MIR222HG using an ASO. β-Actin served as the negative control. **K, L** Co-IP was performed to detect the interaction between H4 and HDAC5 after knockdown (left) or overexpression (right) of MIR222HG in GSC267 cells using anti-H4 **(K)** and anti-HDAC5 **(L)** antibodies, respectively. **M, N** Co-IP was performed to detect the interaction between YWHAE and HDAC5 after knockdown (left) or overexpression (right) of MIR222HG in GSC267 cells using anti-YWHAE **(M)** and anti-HDAC5 **(N)** antibodies, respectively. All data are presented as the means ± SDs. ns, P > 0.05; *P < 0.05; **P < 0.01; ***P < 0.001.

To investigate the mechanism by which nuclear-localized MIR222HG is specifically involved in PMT, we performed RNA-seq of GSC267 cells with MIR222HG knockdown and isolated the proteins bound to MIR222HG by RNA pulldown for analysis by mass spectrometry. GSEA of the RNA-seq data (GEO: GSE213310) indicated that MIR222HG mediates PMT in GSCs at the transcriptome level (Figure 3A and Figure S3A). Intranuclear lncRNAs can be involved in regulating the level of transcription, for example, through histone modifications 30. We screened the mass spectrometry data for nuclear proteins and found that MIR222HG could bind to histone H4 (Figure 3B and Table S2).

However, no histone-modifying enzymes corresponding to histones were identified in the data. We therefore speculated that there may be intermediary proteins involved in histone modifications in the mass spectrometry data. We then predicted interactions with all remaining intranuclear proteins with the STRING database (https://cn.string-db.org/) and found that only YWHAE could bind to the histone-modifying enzyme HDAC5 (Figure 3C). We therefore speculated that MIR222HG may participate in PMT by modifying H4 through the binding of YWHAE to HDAC5. Numerous studies have shown that histone deacetylation is an important factor in promoting tumor development 31, 32. In addition, histone deacetylation has been shown to promote GSC growth and enhance the MES subtype^33, 34^. HDAC5 is an enzyme responsible for histone deacetylation and has not been studied in GSCs. Next, we used GSC267 cells to conduct a series of validations of the putative downstream mechanism. First, we visualized the secondary structures of MIR222HG interacting with YWHAE and H4. We used the RNAfold WebServer (http://rna.tbi.univie.ac.at//cgi-bin/RNAWebSuite/RNAfold.cgi) to predict the secondary structure of MIR222HG and divided it into four main substructures, each containing a base-pairing structure and a hairpin structure. The RNA pulldown assay demonstrated that YWHAE binds to secondary structure 2 (nucleotides 230-750) of MIR222HG and that H4 binds to secondary structure 3 (nucleotides 750-1150) of MIR222HG (Figure 3D). We again validated the binding of MIR222HG to YWHAE and H4 using a RIP assay (Figure 3E). Based on the YWHAE binding site identified in the mass spectrometry results, a plasmid with a deletion mutation was constructed and validated by the RIP assay (Figure S3B). Co-IP confirmed the binding of YWHAE to HDAC5 and the binding of H4 to HDAC5 (Figure 3F, G, I). Western blotting showed no change in YWHAE and HDAC5 expression after knockdown of MIR222HG, and no alteration of YWHAE and HDAC5 expression at the transcriptional level was observed in the RNA-seq results, suggesting that MIR222HG does not affect the intracellular content of YWHAE and HDAC5 (Figure 3J). To determine the specific site where MIR222HG, YWHAE, and HDAC5 cooperate to act on H4, we found that the acetylation level of H4K16 was significantly increased based on Western blotting after knockdown of MIR222HG, indicating that the combined effects of MIR222HG, YWHAE, and HDAC5 mainly led to the deacetylation of H4K16 (Figure 3H). Furthermore, we examined the binding affinity between YWHAE, HDAC5, and H4 by knocking down and overexpressing MIR222HG. The Co-IP results showed that the binding affinity of H4 for HDAC5 was reduced after knockdown of MIR222HG and increased after overexpression of MIR222HG (Figure 3K, L). The binding affinity of YWHAE for HDAC5 did not change significantly after either knockdown or overexpression of MIR222HG, indicating that MIR222HG did not affect the binding of YWHAE and HDAC5 (Figure 3M, N). Taken together, these results suggest that MIR222HG anchors to H4 via secondary structure 3 and then binds to the YWHAE/HDAC5 complex via secondary structure 2, leading to deacetylation of H4K16.

### MIR222HG induces PMT through H4K16 deacetylation, leading to activation of the STAT3 and MAPK pathways

**Figure 4:**
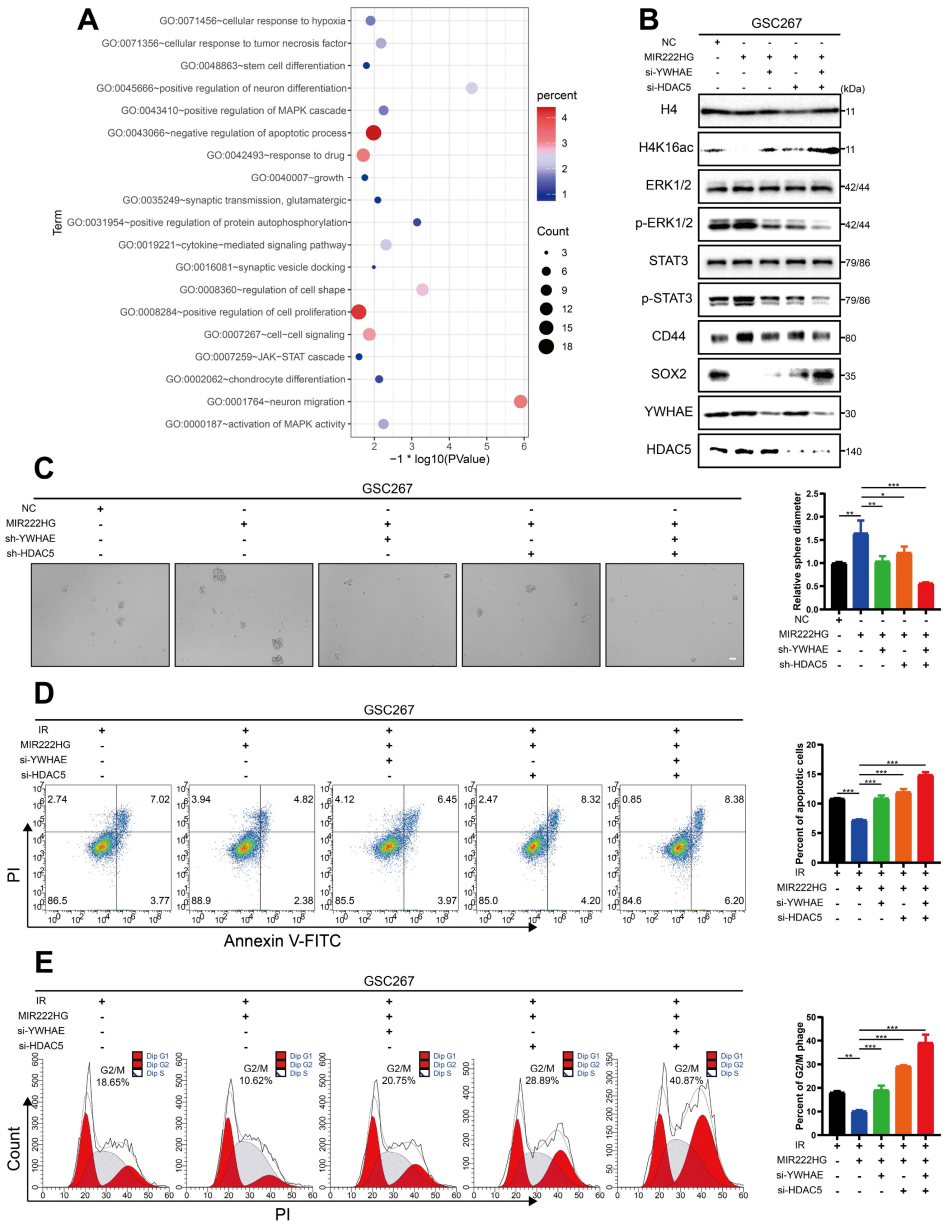
** MIR222HG induces PMT through H4K16 deacetylation, leading to activation of the STAT3 and MAPK pathways. A** The RNA-seq results were used to identify the enriched pathways after interference with MIR222HG expression in GSC267 cells. **B** western blot analysis of the protein levels of H4K16ac, ERK1/2, p-ERK1/2, STAT3, p-STAT3, CD44, SOX2, YWHAE and HDAC5 in GSC267 cells subjected to the indicated interventions. H4 served as the negative control. **C** Tumor sphere formation assays of GSC267 cells subjected to the indicated interventions. The quantification of the relative sphere diameter is shown on the right. Scale bar, 50 μm. **D** Apoptosis assays of GSC267 cells subjected to the indicated interventions. The quantification of apoptosis rates is shown on the right. **E** Cell cycle analysis of GSC267 cells subjected to the indicated interventions. The quantification of the G2/M-phase population is shown on the right. All data are presented as the means ± SDs. ns, P > 0.05; *P < 0.05; **P < 0.01; ***P < 0.001.

Next, we further explored the changes in the downstream pathways following MIR222HG-mediated H4K16 deacetylation via the YWHAE/HDAC5 complex. Enrichment analysis based on previous sequencing results showed that knockdown of MIR222HG resulted in changes in the MAPK and JAK-STAT pathways (Figure 4A and Table S3). The STAT3 pathway has been extensively documented as a classical pathway associated with the MES subtype, and the ERK-MAPK pathway has been previously demonstrated to be a key factor contributing to the MES-like subtype state of GSCs 35, 36. We validated the changes in the downstream pathways by interfering with two key proteins, YWHAE and HDAC5, in the MIR222HG complex. First, we selected the most efficient interference sequence for subsequent experiments (Figure S3E, F). Western blot analysis confirmed that the P-ERK1/2 and P-STAT3 levels were significantly elevated after overexpression of MIR222HG, while knockdown of YWHAE or HDAC5 attenuated these increases in P-ERK1/2 and P-STAT3, and combined knockdown of YWHAE and HDAC5 further reduced the P-ERK1/2 and P-STAT3 levels (Figure 4B and Figure S3C). In addition, overexpression of MIR222HG resulted in enhanced acetylation of H4K16, elevated expression of CD44, and decreased expression of SOX2. Knockdown of YWHAE or HDAC5 partially reversed these changes, and combined knockdown of YWHAE and HDAC5 further reversed the changes in the H4K16ac, CD44, and SOX2 levels (Figure 4B and Figure S3C). GSC self-renewal was enhanced by overexpression of MIR222HG, knockdown of YWHAE or HDAC5 partially reversed this change, and combined knockdown of YWHAE and HDAC5 further reversed this change, as shown by the neurosphere formation assay and ELDA (Figure 4C and Figure S3D, G).

The apoptosis and cell cycle assays demonstrated that the increased resistance of GSCs to radiotherapy after overexpression of MIR222HG was partially reversed by knockdown of YWHAE or HDAC5 and that combined knockdown of YWHAE and HDAC5 further reversed the increase in radioresistance in GSCs (Figure 4D, E and Figure S3H, I). Finally, we performed western blotting on all GSC and NPC cell lines and found that the deacetylation levels in GSCs were higher than those in NPCs, while in GSCs, the deacetylation levels were generally higher in the MES subtype than in the PN subtype (Figure S3J). In summary, MIR222HG mediates the deacetylation of H4K16 via YWHAE/HDAC5, activating the STAT3 and ERK-MAPK pathways, in turn leading to PMT and enhancing radioresistance in GSCs.

### Cotranscription of miR221 and miR222 in GSCs leads to an immunosuppressive state in macrophages via exosomes

**Figure 5:**
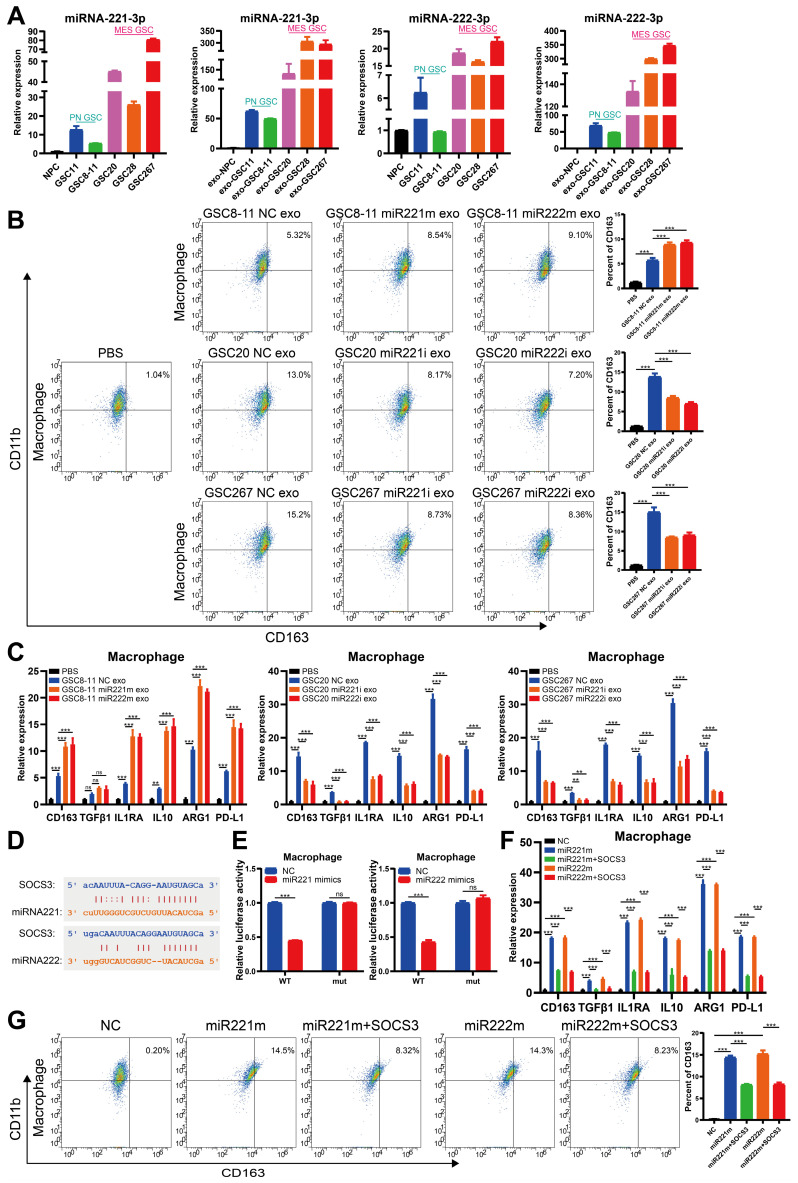
** Cotranscription of miR221 and miR222 in GSCs leads to an immunosuppressive state in macrophages via exosomes. A** qRT‒PCR showing the relative expression of miR221 and miR222 in NPCs, pGSCs (GSC11 and GSC8-11) and mGSCs (GSC20, GSC28, and GSC267) as well as the corresponding exosomes. **B** Representative flow cytometric analysis showing the proportion of CD11b^+^CD163^+^ cells among THP1 differentiated macrophages treated with PBS or exosomes (exo) collected from GSC20 and GSC267 cells transfected with the inhibitor NC, miR221 inhibitor (miR221i) or miR222 inhibitor (miR222i) and GSC8-11 cells transfected with the mimic NC, miR221 mimic (miR221m) or miR222 mimic (miR222m). The histogram shows the proportion of CD11b^+^CD163^+^ cells among THP1 differentiated macrophages. **C** qRT‒PCR assay showing the relative expression of macrophage-derived immunosuppressive genes in THP1 differentiated macrophages treated with PBS or exosomes collected from GSC20 and GSC267 cells transfected with inhibitor NC, miR221i or miR222i and GSC8-11 cells transfected with mimic NC, miR221m or miR222m. **D** The predicted binding site of miR221 and miR222 in the SOCS3 3'UTR. **E** Luciferase activity of SOCS3-3'-UTR WT/mut after transfection with miR221m or miR222m. **F** qRT‒PCR assay showing the relative expression of macrophage-related immunosuppressive genes in THP1 differentiated macrophages cotransfected with the SOCS3 overexpression plasmid (OV-SOCS3) and miR221m or miR222m. **G** Representative flow cytometric analysis showing the proportion of CD11b^+^CD163^+^ cells among THP1 differentiated macrophages cotransfected with OV-SOCS3 and miR221m or miR222m. The histogram shows the proportion of CD11b^+^CD163^+^ cells among THP1 differentiated macrophages. All data are presented as the means ± SDs. ns, P > 0.05; *P < 0.05; **P < 0.01; ***P < 0.001.

It is well documented that TAMs in the microenvironment promote malignant progression of GBM and that GBM in turn promotes immunosuppressive polarization of TAMs 18, 26, 35, 37. Our previous study demonstrated that immunosuppressive M2 macrophages can deliver miR-221-3p, miR-22-3p, and miR-27a-3p to GSCs to promote PMT in GSCs 14. qRT‒PCR revealed that miRNA-221-3p and miRNA-222-3p, cotranscribed from the MIR222HG gene, were not only highly expressed in mGSCs but also detected in their exosomes (Figure 5A). We therefore sought to determine whether mGSCs can affect the macrophage status by delivering miRNA-221-3p and miRNA-222-3p to TAMs via exosomes. We used phorbol 12-myristate 13-acetate (PMA)-treated THP1 cells as macrophages for validation in subsequent experiments. First, we identified exosomes isolated from GSCs by TEM, ZetaView analysis and western blot analysis (Figure S4A, B, C). By uptake experiments, we confirmed that exosomes derived from GSCs can be phagocytosed by macrophages (Figure S4D). Flow cytometric analysis showed that exosomes from GSCs induced the expression of the immunosuppressive macrophage marker CD163 and that the release of exosomes was more strongly induced from mGSCs than from pGSCs. In macrophages, treatment with exosomes derived from GSC8-11 cells overexpressing miR221 and miR222 resulted in elevated CD163 expression compared to treatment with exosomes from untreated GSC8-11 cells. In contrast, treatment with exosomes from GSC20 and GSC267 cells with miR221 and miR222 knockdown resulted in decreased CD163 expression in macrophages compared to treatment with exosomes from untreated GSC20 and GSC267 cells (Figure 5B). In addition, we examined a series of macrophage-derived immunosuppressive genes, including CD163, TGFB1, IL1RA, IL10, ARG1 and PD-L1, in the above treated macrophages by qRT‒PCR and found that the changes in the expression of these genes were generally consistent with those identified by flow cytometry (Figure 5C). This finding demonstrates that miR221 and miR222, which are highly expressed in mGSCs, can act on macrophages via exosomes, leading to immunosuppressive polarization of macrophages. Next, we investigated the specific mechanisms by which miR221 and miR222 act on macrophages. It has been shown that miR221 and miR222 can act on SOCS3 to inhibit angiogenesis in GBM 38. It has also been reported that SOCS3 is associated with central nervous system (CNS) immunity as a tumor suppressor and that inhibition of SOCS3 leads to immunosuppressive M2 polarization of macrophages 39, 40. We analyzed the miR221 and miR222 sequences with ENCORI (https://starbase.sysu.edu.cn/index.php) and found that they share common binding sites for the SOCS3 3′ untranslated region (UTR) (Figure 5D). We validated the SOCS3 binding sites in miR221 and miR222 by a luciferase reporter assay (Figure 5E). Western blot analysis showed that overexpression of miR221 and miR222 in macrophages reduced SOCS3 expression (Figure S4E). Flow cytometric analysis of macrophages with direct overexpression of miR221 and miR222 followed by further overexpression of SOCS3 showed that overexpression of miR221 and miR222 increased CD163 expression in macrophages and that further overexpression of SOCS3 partially reversed the increase in CD163 expression caused by overexpression of miR221 and miR222 (Figure 5G). qRT‒PCR showed changes in the levels of immunosuppressive genes, including CD163, TGFB1, IL1RA, IL10, ARG1 and PD-L1, in these treated macrophages, in general agreement with the flow cytometry results (Figure 5F). Finally, we validated the downstream pathway through which miR221 and miR222 affect immunosuppressive macrophage polarization via SOCS3. Previous studies have demonstrated that knockdown of SOCS3 leads to STAT3 activation and that NF-κB inhibition causes M2 polarization of macrophages 40. We found that overexpression of miR221 and miR222 resulted in elevated P-STAT3 and decreased P-P65 levels, while further overexpression of SOCS3 reversed the changes induced by overexpression of miR221 and miR222 (Figure S4E). Taken together, these results demonstrate that miR221 and miR222, which are highly expressed in mGSCs, can act via exosomes on SOCS3 in macrophages, altering the STAT3 and NF-κB pathways and leading to immunosuppressive polarization of macrophages.

### The transcription factor SPI1 regulates cotranscription of the MIR222HG gene

**Figure 6:**
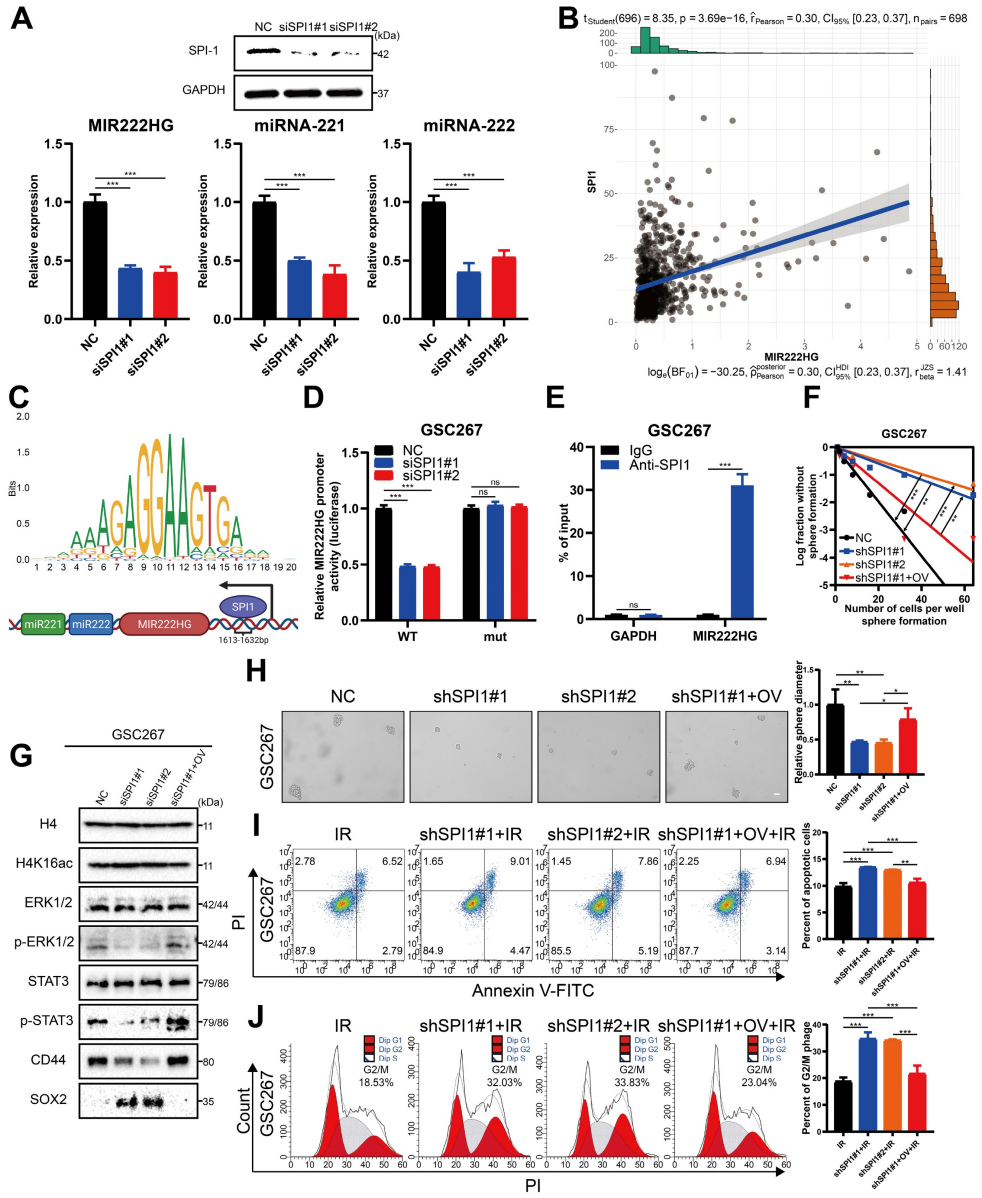
** The transcription factor SPI1 regulates cotranscription of the MIR222HG gene. A** Detection of SPI1 expression by western blotting and miR222HG, miR221 and miR222 expression by qRT‒PCR after knockdown of SPI1 in GSC267 cells. **B** Positive correlation between SPI1 expression and MIR222HG expression. **C** Predicted SPI1-binding sites in the promoter region of MIR222HG. **D** Luciferase activity of the MIR222HG promoter after transfection with siSPI1#1 and siSPI1#2. **E** ChIP assay of the enrichment of SPI1 on the MIR222HG promoter region. **F** ELDA of GSC267 cells subjected to the indicated interventions. **G** western blot analysis of the protein levels of H4K16ac, ERK1/2, p-ERK1/2, STAT3, p-STAT3, CD44 and SOX2 in GSC267 cells subjected to the indicated interventions. H4 served as the negative control. **H** Tumor sphere formation assays of GSC267 cells subjected to the indicated interventions. The quantification of the relative sphere diameter is shown on the right. Scale bar, 50 μm. **I** Apoptosis assay of GSC267 cells subjected to the indicated interventions. The quantification of apoptosis rates is shown on the right. **J** Cell cycle analysis of GSC267 cells subjected to the indicated interventions. The quantification of the G2/M-phase population is shown on the right. All data are presented as the means ± SDs. ns, P > 0.05; *P < 0.05; **P < 0.01; ***P < 0.001.

Next, we sought to identify common upstream targets that regulate the expression of MIR222HG, miR221, and miR222. Using JASPAR (https://jaspar.genereg.net/), we predicted transcription factors for the MIR222HG gene and initially identified 11 potential transcription factors. The relationships of these transcription factors with prognosis, subtype and immune characteristics were then analyzed by a bioinformatic approach, and we eventually targeted the transcription factor SPI1. SPI1, an immune-related oncogenic factor, was previously studied mostly in leukaemia 41. A recent study by our group found that SPI1 acts as a transcription factor that promotes GBM progression and that knocking down SPI reduces CD44 expression 42. GSEA based on the Verhaak and Phillips gene sets showed that high expression of SPI1 was found to be more prevalent in the MES subtype and low expression of SPI1 was more prevalent in the PN subtype (Figure S5A, B). Based on marker gene correlation analysis, SPI1 expression was positively correlated with the expression of MES subtype marker genes (CD44, CHI3L1 and SERPINE1) and negatively correlated with the expression of PN subtype marker genes (DLL3, OLIG2, ASCL1, NCAM1) (Figure S5C and Table S4). SPI1 was highly expressed in the MES subtype in both the TCGA and CGGA databases, and high expression of SPI1 was associated with a poor prognosis (Figure S5D, E). Single-cell RNA-seq analysis showed that clusters of cells with high SPI1 expression had higher MES scores (Figure S5F). Moreover, high SPI1 expression in GBM was associated with multiple immune features and pathways (Figure S5G, H and Table S5). Sequence analysis of the SPI1, a possible transcription factor for MIR222HG, revealed that it has only one binding site located upstream of the MIR222HG gene from positions 1613-1632 (Figure 6C). Correlation analysis revealed that MIR222HG expression was positively correlated with SPI1 expression (Figure 6B). The luciferase reporter and the ChIP assays demonstrated that SPI1 promotes MIR222HG transcription by binding to positions 1613-1632 upstream of the MIR222HG gene (Figure 6D, E). The expression of MIR222HG, miR221 and miR222 was downregulated after SPI1 knockdown (Figure 6A). Next, we found that knockdown of SPI1 decreased the self-renewal ability of GSC20 and GSC267 cells, while overexpression of MIR222HG reversed this change (Figure 6F, H and Figure S6A, B). The reduction in radioresistance in GSC20 and GSC267 cells after knockdown of SPI1 was reversed after overexpression of MIR222HG (Figure 6I, J and Figure S6D, E). Western blot analysis showed that knockdown of SPI1 increased H4K16 acetylation levels, decreased ERK1/2 and STAT3 phosphorylation levels, increased SOX2 expression and decreased CD44 expression in GSC20 and GSC267 cells, while overexpression of MIR222HG reversed these changes (Figure 6G and Figure S6C). In conclusion, these findings demonstrate that SPI1 acts as a transcription factor that mediates cotranscription of MIR222HG, miR221 and miR222, thereby causing downstream changes.

### Targeting mGSCs with the SPI1 expression-sensitive drug PLX-4720 in combination with radiotherapy

**Figure 7:**
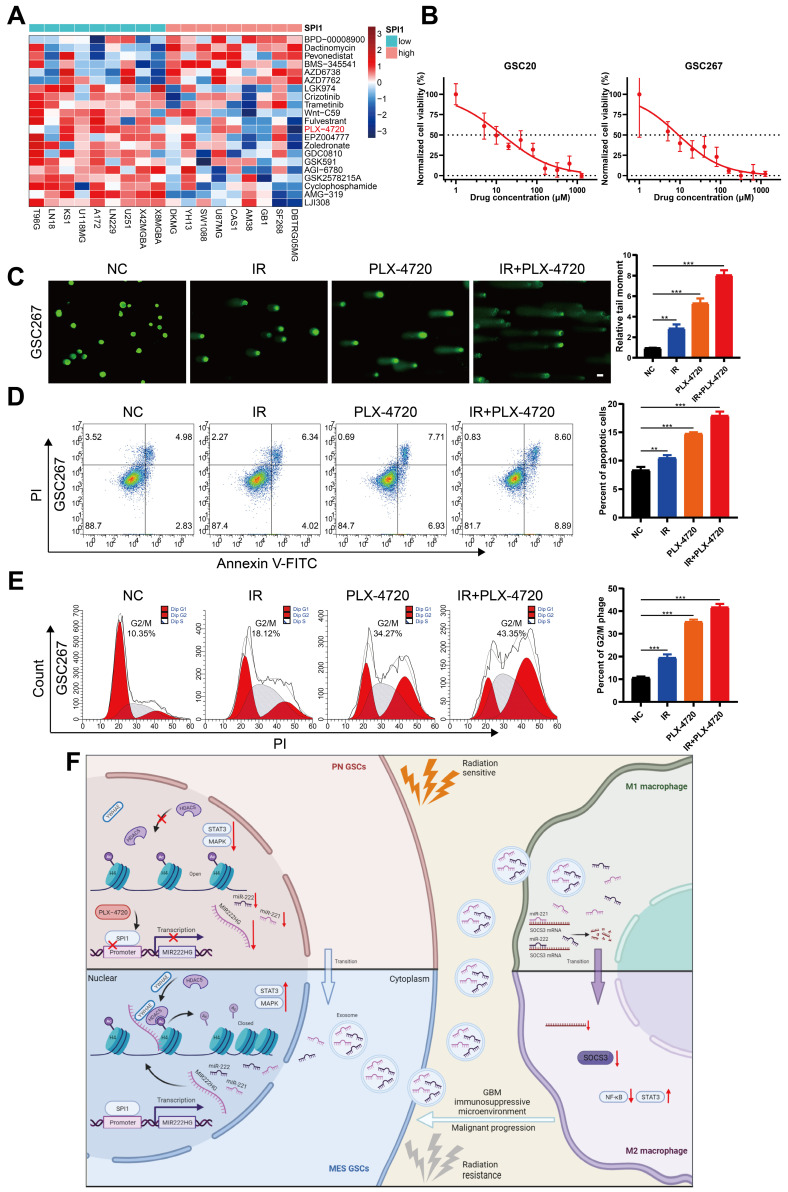
** Targeting mGSCs with the SPI1 expression-sensitive drug PLX-4720 in combination with radiotherapy. A** Heatmap visualizing the differences in the sensitivity of cell lines with high and low expression of SPI1 to the indicated drugs. **B** CCK-8 assay of GSC20 and GSC267 cells treated with different concentrations of PLX-4720 for 48 hours. **C** Comet assays of GSC267 cells treated with IR (6 Gy) and PLX-4720. The quantification of the relative tail moment is shown on the right. Scale bar, 25 μm. **D** Apoptosis assay of GSC267 cells treated with IR (6 Gy) and PLX-4720. The quantification of apoptosis rates is shown on the right. **E** Cell cycle analysis of GSC267 cells treated with IR (6 Gy) and PLX-4720. The quantification of the G2/M-phase population is shown on the right. **F** Proposed working model of the function of the MIR222HG gene in PMT in GSCs and immunosuppressive polarization of macrophages. All data are presented as the means ± SDs. ns, P > 0.05; *P < 0.05; **P < 0.01; ***P < 0.001.

As SPI1, which mediates MIR222HG transcription, is highly expressed in the MES subtype, we investigated the relationship between SPI1 expression and therapeutic drug sensitivity. By screening for drugs to which GBM cell lines with high SPI1 expression are sensitive and considering factors such as the blood‒brain barrier, PLX-4720 was finally identified (Figure 7A and Table S6). PLX-4720 is a B-Raf inhibitor that has been documented to exert anti-GBM effects *in vitro* and *in vivo* in combination with radiotherapy 43. We determined the IC50 of PLX-4720 in GSC20 and GSC267 cells by a CCK-8 assay (Figure 7B). By treating GSC20 and GSC267 cells with PLX-4720 or radiotherapy, we found that PLX-4720 had better efficacy than radiotherapy alone, resulting in increased DNA damage, increased apoptosis, and significant G2/M arrest. The combination of PLX-4720 and radiotherapy resulted in further increases in DNA damage and apoptosis and further G2/M arrest in GSCs (Figure 7C, D, E and Figure S6F, G, H). These results show that PLX-4720 has a significant therapeutic effect in radioresistant mGSCs.

## Discussion

In recent years, as the study of noncoding RNAs has intensified, an increasing number of researchers have discovered that the transcribed sequences of some miRNAs are located in lncRNA host genes, or MIRHGs 44. Initially, researchers focused on the function of miRNAs transcribed from host genes, but researchers are increasingly turning their attention to the lncRNAs produced from MIRHGs 44-47. Host genes from which miRNAs and lncRNAs are coexpressed can synergistically influence tumor progression. In this study, we demonstrated the host gene for miR221 and miR222:MIR222HG. MIR222HG, also called Lnc-Ang362, has been studied more frequently in cardiovascular disease than in other processes and can promote smooth muscle cell proliferation 48. In tumors, MIR222HG has been reported to promote the development of prostate cancer 49. In this study, the MIR222HG gene was found to function in GBM primarily through the production of the lncRNA transcript MIR222HG-201, which is localized in the nucleus. MIR222HG is regulated by the transcription factor SPI1 and mediates H4K16 deacetylation via YWHAE-bound HDAC5, facilitating PMT and enhancing radioresistance in GSCs. miR221 and miR222 cotranscribed with MIR222HG-201 can act via exosomes on SOCS3 in macrophages and cause immunosuppressive polarization of macrophages. Finally, the SPI1 expression-sensitive drug PLX-4720 can be used in combination with radiotherapy to target mGSCs (Figure 7F). In conclusion, the MIR222HG gene promotes malignant progression and treatment resistance in GBM by affecting both GSCs themselves and macrophages in the microenvironment.

The high inter- and intratumoural heterogeneity of GBM and the inherent plasticity of GBM cells are challenges that lead to therapeutic resistance in GBM and impede progress in clinical treatment 50. GBM heterogeneity and plasticity arise mainly from the different subtypes of GSCs and their interactions with the complex tumor microenvironment 4. GBM recurrence and multitreatment resistance are thought to be associated with PMT, and the exploration of PMT mechanisms is a current research focus. Bhat et al suggested that PN GSCs can be induced to transition into the MES state in an NF-κB-dependent manner, with associated enrichment of CD44-expressing cells and an increase in radioresistance 7. Zhengxin Chen et al. suggested that FOSL1 promotes PMT in GSCs via the UBC9/CYLD/NF-κB axis 51. HDAC inhibitors, as antitumor agents, have been reported in extensive studies, including studies in GBM 52. Melissa M Singh et al. combined a pan-HDAC inhibitor and a KDM1A inhibitor for the treatment of GSCs 53. HDAC1 has also been reported to play an important role in the maintenance and transition of the MES subtype in GSCs via the NF-κB pathway 34. In this study, we showed that MIR222HG in GSCs can affect the dual STAT3 and MAPK pathway-mediated MES transition through H4K16 deacetylation by YWHAE-bound HDAC5, resulting in malignant progression and radioresistance in GBM. In addition, deacetylation of H4K16 is considered a hallmark in human cancers, and its role in GBM was confirmed in our study 54.

The interaction of the tumor immune microenvironment with GBM cells is an important factor contributing to GBM heterogeneity and the resulting therapeutic resistance. It was previously reported that macrophages can mediate the MES-like state of GBM via OSM/OSMR 35. Recent studies have shown that PMT in GBM is accompanied by changes in both the intrinsic anatomical location of the tumor and the microenvironment, including immunity 18. Our previous study demonstrated that M2 macrophages can cause PMT through delivery of miR221, etc., to GSCs 14. In this study, we explored whether mGSCs can mediate immunosuppressive polarization through the delivery of miR221 and miR222 cotranscribed from MIR222HG to macrophages. We demonstrated that GSCs and macrophages can interact through the delivery of various substances that together promote malignant tumor progression. Xiaomeng Li et al. previously reported that the expression of MIR222HG, an immune-related lncRNA, was positively correlated with B7-H3 and PDL1 expression, as determined by bioinformatics analysis 55. This corroborates our findings that immune-related lnc-MIR222HG, which is highly expressed in mGSCs, can produce cotranscribed miRNAs for exosomal delivery and in turn cause macrophage immunosuppression and elevated PDL1 expression (Figure 5C). Since we did not detect nuclear lnc-MIR222HG expression in exosomes in our previous study, it is certain that the immunoregulatory function of the MIR222HG gene is performed through miR221 and miR222. Immune checkpoint inhibitors, such as anti-PD1/PDL1 antibodies, have achieved satisfactory results in the treatment of several cancer types, and Hao Zhang et al. showed that the expression of lncRNAs in GBM can be used to assess responsiveness to immune checkpoint therapies 56, 57. Based on these findings and the findings of this study, we will explore the role of the MIR222HG gene in anti-PD1/PDL1 therapy for GBM in a subsequent study. SOCS3 is considered a tumor suppressor and has been studied in a variety of cancers, and it has been shown to be associated with CNS immunity and to mediate macrophage polarization 39, 40. Xin-Chao Ji et al. showed that a reduction in SOCS3 expression in intracerebral hemorrhage induced M2 macrophage polarization through activation of STAT3 and inhibition of NF-κB 40. Chun-Hua Xu et al. demonstrated that SOCS3 expression could be inhibited by miR221 and miR222 to cause angiogenesis in GBM 38. In this study, we focused on whether miR221 and miR222 cause immunosuppressive polarization of macrophages in GBM through activation of STAT3 and inhibition of NF-κB, which in turn promote malignant progression of GBM.

SPI1, also called PU.1, is an ETS-domain transcription factor that activates gene expression during myeloid and B-lymphoid cell development 58. In tumors, SPI1 was initially studied more frequently in leukemia, but SPI1 was later reported to play a promoting role in other cancers 59-61. For example, our previous study demonstrated that SPI1 promotes GBM progression by regulating FTO and that knockdown of SPI1 leads to a decrease in CD44 expression 42. In this study, we found that the transcription factor SPI1 regulates the expression of the MIR222HG gene, thereby affecting the GSC and macrophage status. Since we detected decreased expression of miR221 and miR222 in GSCs with SPI1 knockdown, we did not further observe the effect of exosomes from SPI1 knockdown GSCs on macrophages (Figure 6A). PLX-4720, a B-Raf inhibitor, has been used in combination with radiotherapy for the treatment of GBM *in vivo* and *in vitro* 43. In the present study, we found that GBM cell lines with high SPI1 expression were more sensitive to PLX-4720, and we validated the combined effect of PLX-4720 and radiotherapy on GSCs by *in vitro* experiments using mGSCs. However, the relationship between the MES subtype and B-Raf needs to be further investigated.

## Conclusions

SPI1-regulated nuclear lnc-MIR222HG-201 in GSCs anchors H4 through the binding of YWHAE to HDAC5 to mediate H4K16 deacetylation in order to activate the STAT3 and MAPK pathways, thereby causing PMT and enhancing radioresistance in GSCs. Cotranscribed miR221 and miR222 act on macrophages via exosomes, leading to downregulation of SOCS3, activation of the STAT3 pathway, and inhibition of the NF-κB pathway, resulting in immunosuppressive polarization of macrophages. The SPI1 expression-sensitive drug PLX-4720 has a significant therapeutic effect on radioresistant mGSCs. Our findings indicate that targeting the MIR222HG gene may be a promising approach to preventing PMT, activating immunity and overcoming treatment resistance in GBM.

## Supplementary Material

Supplementary figures.

Supplementary tables.

## Abbreviations

ASO: antisense oligonucleotide
CCK-8: cell counting kit-8
CGGA: chinese glioma genome atlas
ChIP: chromatin immunoprecipitation
CL: classical
CO-IP: co-immunoprecipitation
ECM: extracellular matrix
ELDA: extreme limiting dilution assay
ENA: european nucleotide archive
FISH: fluorescence *in situ* hybridization
GBM: glioblastoma
GSCs: glioma stem cells
IHC: immunohistochemical
IR: ionizing radiation
LncRNAs: Long non-coding RNAs
MES: mesenchymal
mGSCs: mesenchymal glioblastoma stem cells
MIRHG: micro RNA host gene
miRNA: micro RNA
miR: micro RNA
NPC: neural progenitor cell
pGSCs: proneural glioblastoma stem cells
PMT: proneural-to-mesenchymal transition
PN: proneural
qRT-PCR: real-time quantitative PCR
RIP: RNA immunoprecipitation
sEVs: small extracellular vesicles
TAM: tumor-associated macrophages
TCGA: the cancer genome atlas
TME: tumor microenvironment
TUNEL: transferase dUTP nick end labeling
