# Supplementary material for: SPI1-mediated MIR222HG transcription promotes proneural-to-mesenchymal transition of glioma stem cells and immunosuppressive polarization of macrophages
Source: Theranostics. 2023 May 27;13(10):3310–29. doi: 10.7150/thno.82590 (PMC10283056; doi:10.7150/thno.82590)
Supplement: Supplementary file 1 — Supplementary figures. [file thnov13p3310s1.pdf]

Supplementary Figures

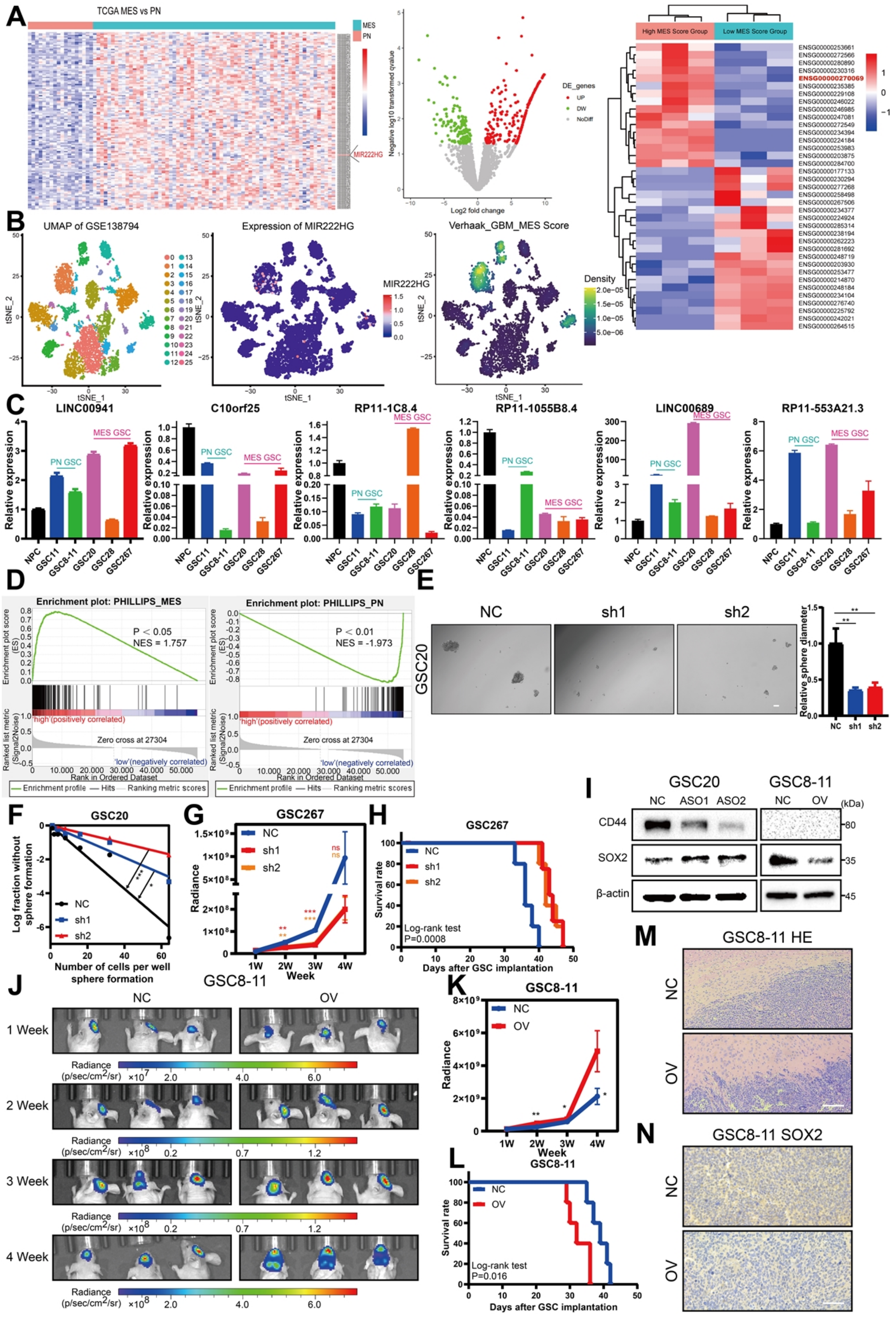

Figure S1. A Heatmap of LncRNAs that were diferentially expressed between MES GBM and PN GBM based on TCGA (left) and Qilu dataset (right). Volcano plots of LncRNAs that

were diferentially expressed between MES GSCs and PN GSCs based on ENA (medium). **B** Single-cell RNA sequencing of GSE138794 visualizing UMAP cell clusters, Verhaak_GBM_MES score and MIR222HG expression. **C** qRT-PCR showing the relative expression of the six LncRNAs other than MIR222HG in NPC, pGSCs (GSC11; GSC8-11) and mGSCs (GSC20; GSC28; GSC267). **D** GSEA showed that MIR222HG expression was positively correlated with MES subtype and negatively correlated with PN subtype, based on the Phillips gene set. **E** Representative images and quantification of tumor sphere formation in GSC20 with knocked down MIR222HG. Scale bar, 50 μm. **F** ELDA of GSC20 expressing lentiviral shNC or shMIR222HG. **G** Quantification of tumor size in shNC, sh1 and sh2 GSC267 xenograft nude mice. **H** Kaplan–Meier curves visualizing the survival of GSC267 xenograft mice in different groups. **I** Western blotting assay of CD44 and SOX2 protein expression after knockdown of MIR222HG in GSC20 and overexpression of MIR222HG in GSC8-11. β-actin served as the negative control. **J, K** Bioluminescence images **(J)** and quantification **(K)** of tumor size in vector (NC) and MIR222HG (OV) GSC8-11 xenograft nude mice. **L** Kaplan–Meier curves visualizing the survival of GSC8-11 xenograft mice in different groups. **M** Represented images of H&E staining for a subgroup of animal sacrificed simultaneously in each group. Scale bar, 200 μm. **N** Representative images of IHC staining for SOX2 in sections of GSC8-11 xenografts in each group. Scale bar, 25 μm. All data are presented as the means ± SD, ns, P > 0.05, *P < 0.05, **P < 0.01, ***P < 0.001.

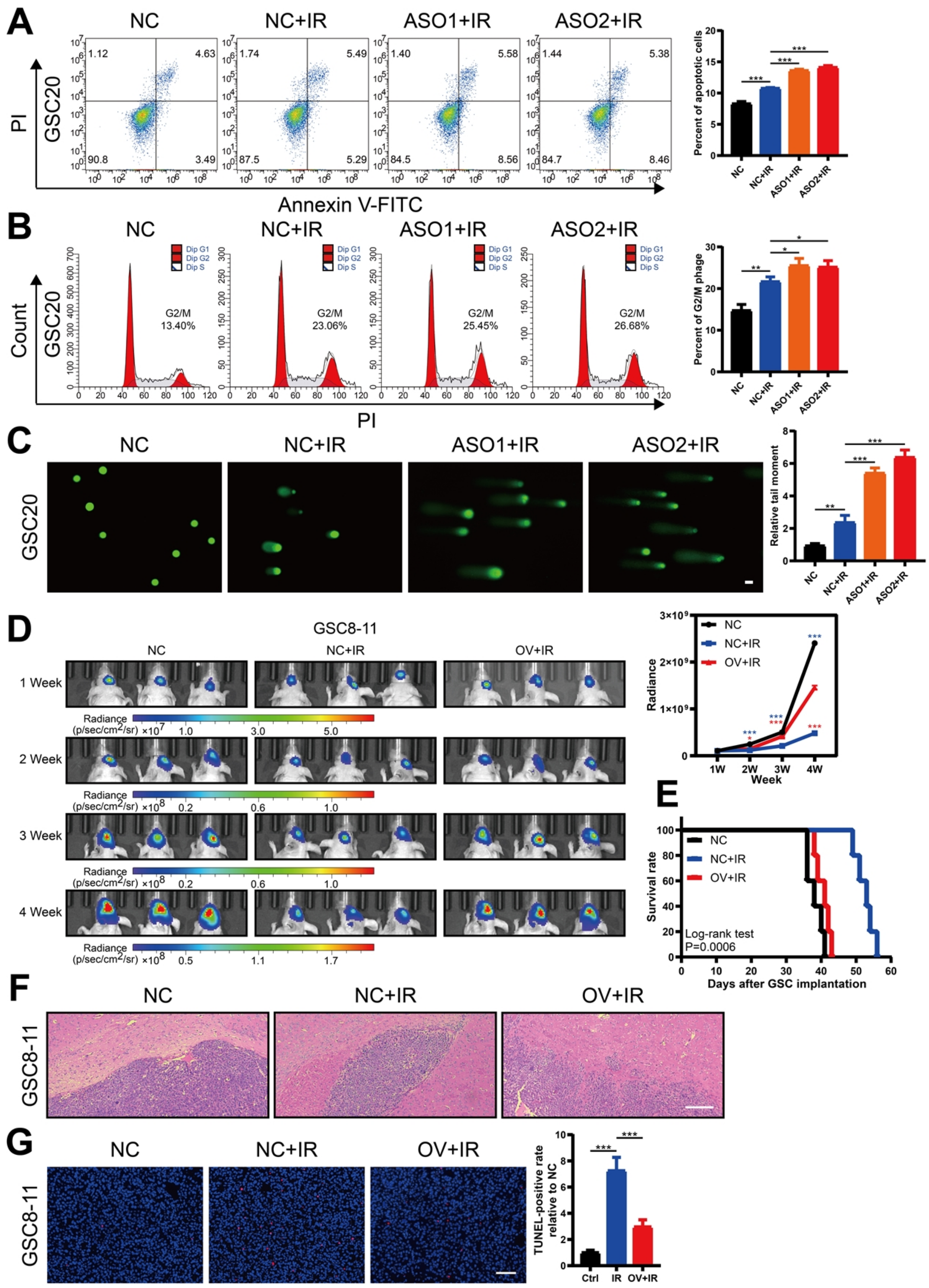

Figure S2. A, B, C Detecting the effect of knocking down MIR222HG in GSC20 on IR (6Gy) treatment by apoptosis **(A)**, cell cycle **(B)** and comet **(C)** assay. The corresponding quantification is shown on the right. Scale bar, 25 μm. **D** Bioluminescence images (left) and quantification (right) of tumor size in vector (NC) and MIR222HG (OV) GSC8-11 xenograft nude mice receiving IR treatment. **E** Kaplan–Meier curves visualizing the survival of GSC8-11 xenograft mice in different groups. **F** Represented images of H&E staining for

a subgroup of animal sacrificed simultaneously in each group. Scale bar, 200 μm. **G** Representative images and quantification of TUNEL staining in sections of IR-treated GSC8-11 xenografts. Scale bar, 200 μm. All data are presented as the means ± SD, ns, $P > 0.05$, *$P < 0.05$, **$P < 0.01$, ***$P < 0.001$.

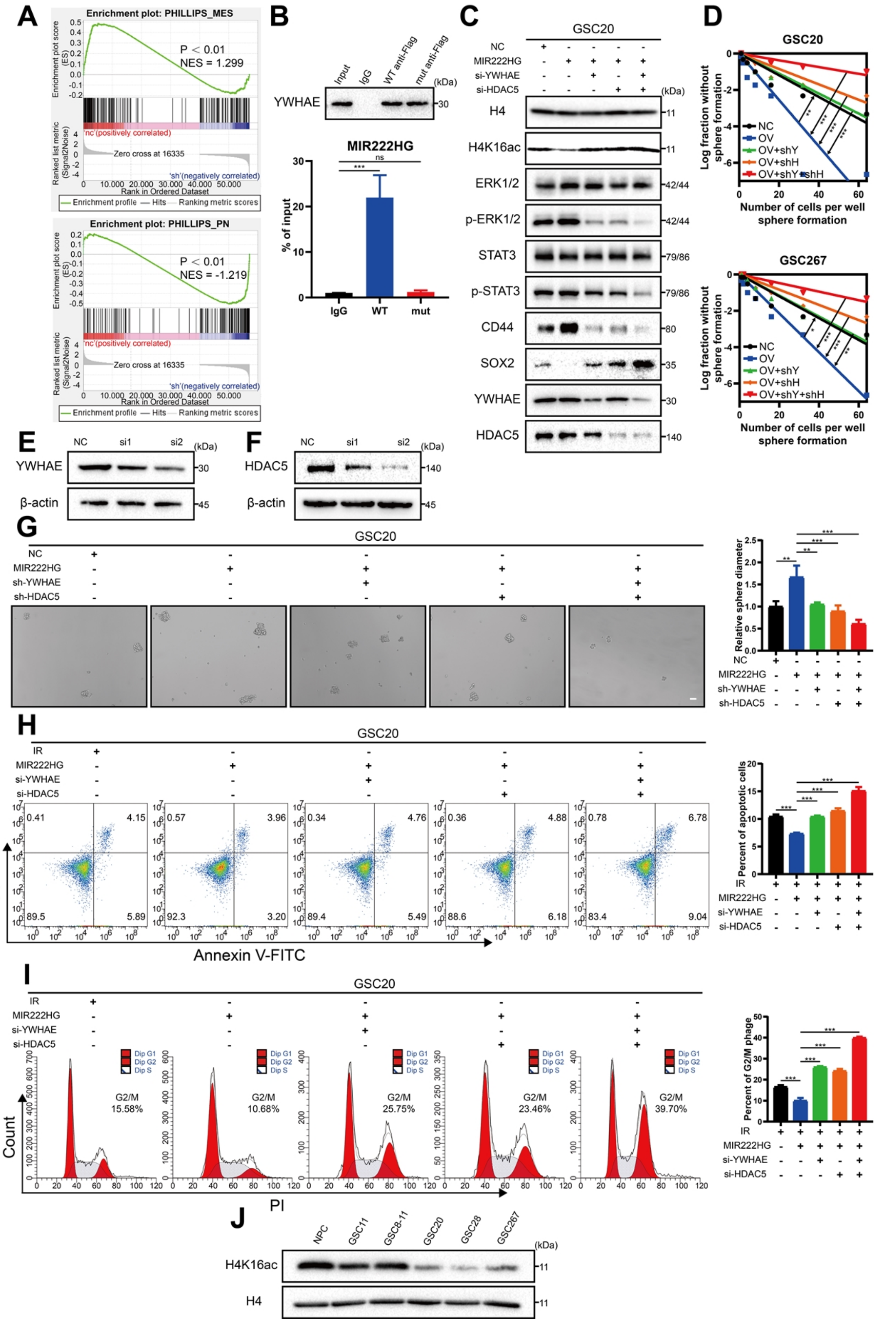

Figure S3. A GSEA showed that knockdown of MIR222HG expression in GSC267 was negatively correlated with the MES subtype and positively correlated with the PN subtype, according to the Phillips gene set. **B** RIP-qPCR assays showing the relative enrichment of MIR222HG WT/mut in GSC267 detected by Flag antibody (bottom). Specific

immunoprecipitation of Flag was confirmed by Western blot (top). **C** Western blotting analysis of protein expression of H4K16ac, ERK1/2, p-ERK1/2, STAT3, p-STAT3, CD44, SOX2, YWHAE and HDAC5 in GSC20 treated with the indicated interventions. H4 served as the negative control. **D** ELDA assays of GSC20 and GSC267 treated with the indicated interventions. **E** Knockdown efficiency of the siRNA of YWHAE in GSC267. **F** Knockdown efficiency of the siRNA of HDAC5 in GSC267. **G** Tumor sphere formation assays of GSC20 treated with the indicated interventions. The quantification of the relative sphere diameter is shown on the right. Scale bar, 50 μm. **H** Apoptosis assays of GSC20 treated with the indicated interventions. The quantification of the apoptosis rates is shown on the right. **I** Cell cycle assays of GSC20 treated with the indicated interventions. The quantification of the G2/M phage rates is shown on the right. **J** Western blotting showing the Level of acetylation of H4K16 in NPC, pGSCs (GSC11; GSC8-11) and mGSCs (GSC20; GSC28; GSC267). H4 served as the negative control. All data are presented as the means ± SD, ns, $P > 0.05$, *$P < 0.05$, **$P < 0.01$, ***$P < 0.001$.

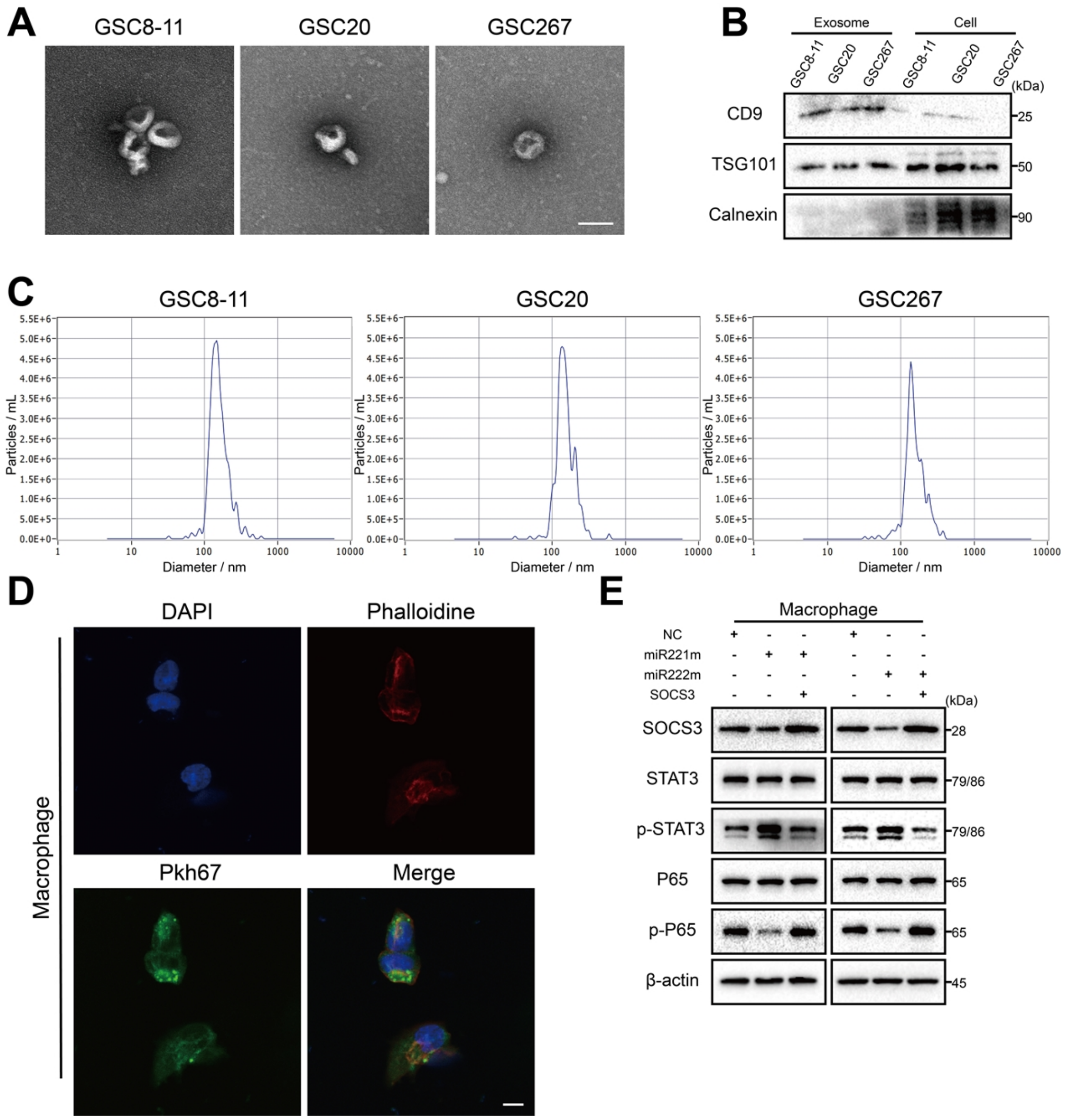

Figure S4. A Representative electron micrographs of exosomes isolated from supernatants of GSCs using differential centrifugation reveal the typical morphology and size of exosomes. Scale bar, 100 nm. **B** Western blot analysis showing the presence of the known exosome markers TSG101 and CD9, and the absence of the negative marker Calnexin in isolated exosomes. **C** ZetaView system was used to analyze exosome particle size. **D** Representative confocal microscopy images reveal the internalization of PKH67-labeled exosomes (green) by THP1 differentiated macrophages. Scale bar, 10 μm. **E** Western blotting analysis of protein expression of SOCS3, STAT3, p-STAT3, P65 and p-P65 in THP1 differentiated macrophages treated with the indicated interventions. β-actin served as the negative control.

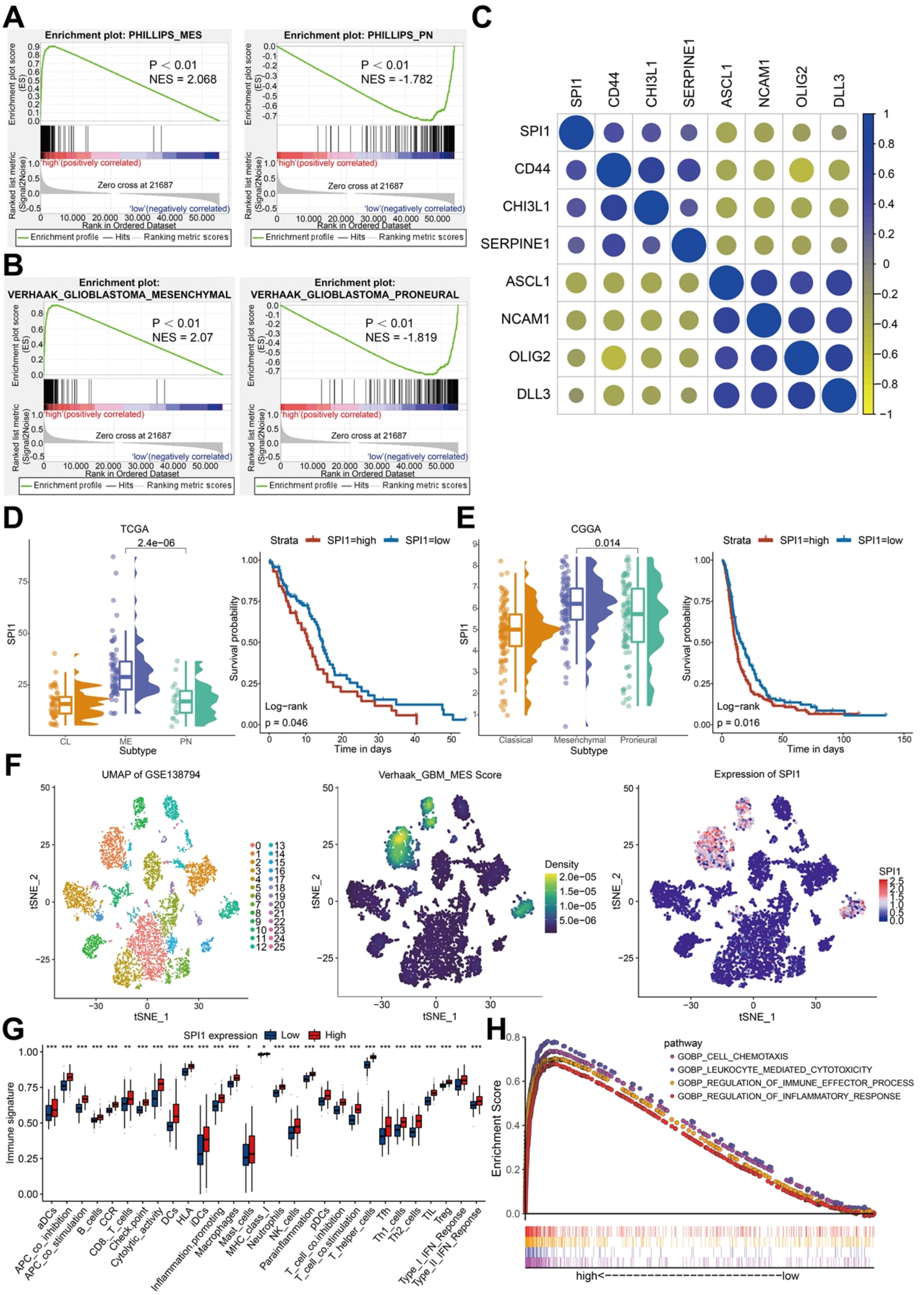

Figure S5. A, B GSEA showed that SPI1 expression was positively correlated with MES subtype and negatively correlated with PN subtype, based on the Phillips **(A)** and Verhaak **(B)** gene set. **C** The correlation of SPI1 expression with PN subtype marker genes (DLL3, OLIG2, ASCL1, NCAM1) and MES subtype marker genes (CD44, CHI3L1 and SERPINE1). **D** The expression of SPI1 in mesenchymal (MES), proneural (PN), and classical (CL)

subtypes in TCGA GBM (left). Kaplan–Meier curves revealing overall survival of TCGA GBM patients stratified in accordance with SPI1 expression (right). **E** The expression of SPI1 in mesenchymal (MES), proneural (PN), and classical (CL) subtypes in CGGA GBM (left). Kaplan–Meier curves revealing overall survival of CGGA GBM patients stratified in accordance with SPI1 expression (right). **F** Single-cell RNA sequencing of GSE138794 visualizing UMAP cell clusters, Verhaak_GBM_MES score and SPI1 expression. **G** The relative abundance of each immune signature in GBM with high and low expression of SPI1. **H** The immune pathways associated with SPI1 expression in GBM. All data are presented as the means ± SD, ns, $P > 0.05$, $^{*}P < 0.05$, $^{**}P < 0.01$, $^{***}P < 0.001$.

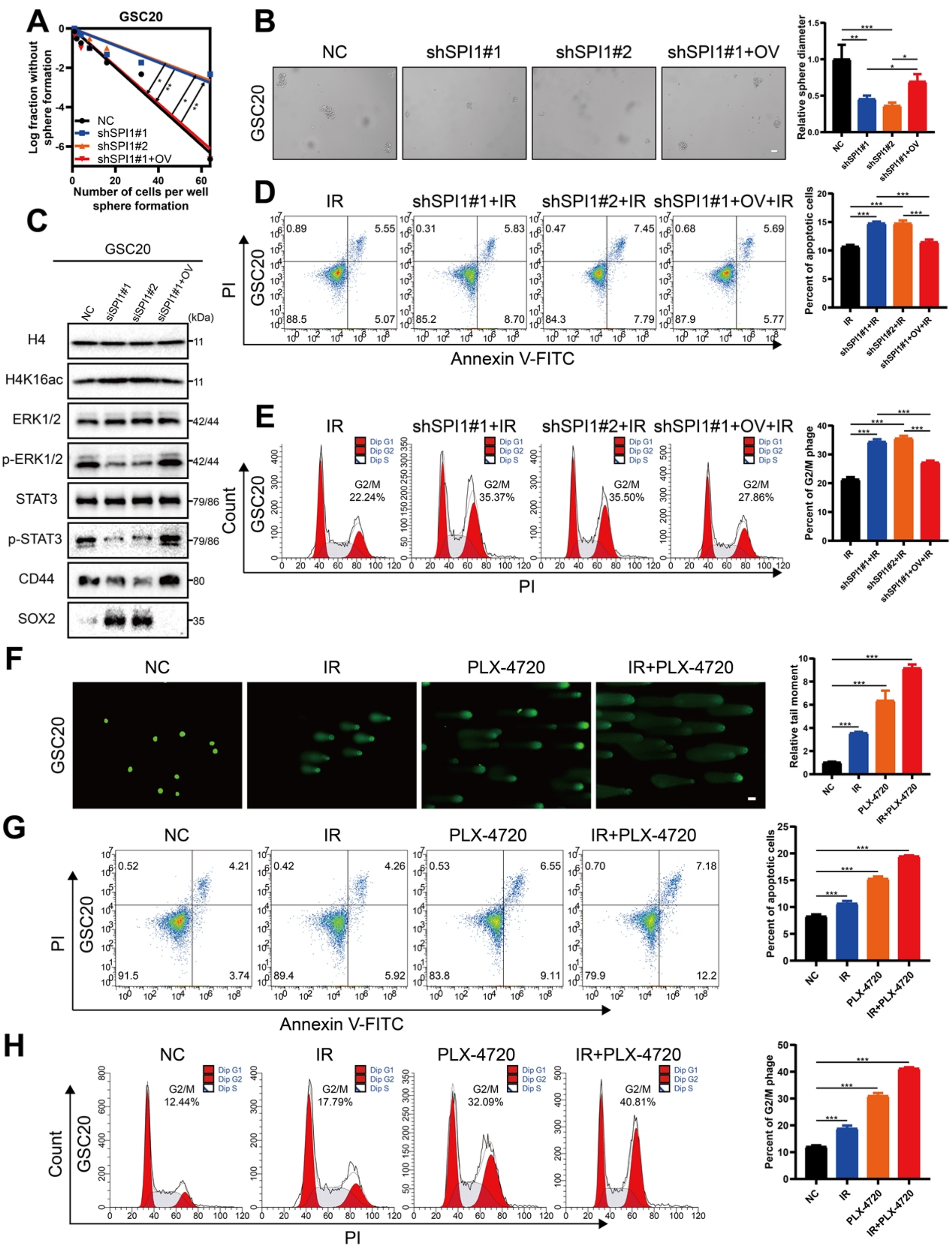

Figure S6. A ELDA assays of GSC20 treated with the indicated interventions. **B** Tumor sphere formation assays of GSC20 treated with the indicated interventions. The quantification of the relative sphere diameter is shown on the right. Scale bar, 50 μm. **C** Western blotting analysis of protein expression of H4K16ac, ERK1/2, p-ERK1/2, STAT3, p-STAT3, CD44 and SOX2 in GSC20 treated with the indicated interventions. H4 served as the negative control. **D** Apoptosis assays of GSC20 treated with the indicated interventions. The quantification of the apoptosis rates is shown on the right. **E** Cell cycle assays of GSC20 treated with the indicated interventions. The quantification of the G2/M

phage rates is shown on the right. **F** Comet assays of GSC20 treated with IR (6 Gy) and PLX-4720. The quantification of the relative tail moment is shown on the right. Scale bar, 25 μm. **G** Apoptosis assays of GSC20 treated with IR (6 Gy) and PLX-4720. The quantification of the apoptosis rates is shown on the right. **H** Cell cycle assays of GSC20 treated with IR (6 Gy) and PLX-4720. The quantification of the G2/M phage rates is shown on the right. All data are presented as the means ± SD, ns, $P > 0.05$, *$P < 0.05$, **$P < 0.01$, ***$P < 0.001$.
